# Effectiveness of an educational intervention to promote psychosocial well-being of school-going adolescents in Sri Lanka

**DOI:** 10.1186/s12889-023-17023-6

**Published:** 2023-11-07

**Authors:** Chiranthika Vithana, Ayesha Lokubalasooriya, Ganeshamoorthy Pragasan, Kanchana Lanka Mahagamage, Kumudumalee Nanayakkara, Himali Prasangika Herath, Priyani Karunarathna, Nadeeka Perera, Chithramalee de Silva, Dushyanthi Jayawardene, Nuwan Darshana Wickramasinghe

**Affiliations:** 1 Family Health Bureau, Ministry of Health, Colombo, Sri Lanka; 2 Base Hospital Horana, Horana, Sri Lanka; 3 National Institute of Health Sciences, Kalutara, Sri Lanka; 4 Department of Community Medicine, Faculty of Medicine, University of Colombo, Colombo, Sri Lanka; 5 Department of Community Medicine, Faculty of Medicine and Allied Sciences, Rajarata University of Sri Lanka, Anuradhapura, Sri Lanka

**Keywords:** Adolescents, Psychosocial well-being, Educational intervention, School-based intervention

## Abstract

**Background:**

One-fifth of the Sri Lankan population consists of adolescents, with 71% of them schooling. An extreme need exists in the country for the introduction of evidence-based interventions for the psychosocial well-being of adolescents. The present study assessed the effectiveness of an educational intervention to promote the psychosocial well-being of school-going adolescents in grade nine in Western Province, Sri Lanka.

**Materials and methods:**

A quasi-experimental study was conducted among grade nine students in Western Province in 2019. Panadura Medical Officer of Health (MOH) area was selected as the interventional area (IA), and Kelaniya MOH area was identified as the control area (CA). Teachers at schools in the IA received training on psychosocial health promotion of adolescents. They delivered the activity-based educational intervention package to the grade nine students as 20-min classroom sessions for three months. Pre- and post-intervention assessments of attitudes and practices related to the psychosocial well-being of adolescents were conducted using an interviewer-administered questionnaire. Categorical data were compared using Chi-Square or Fisher’s exact test. Mann–Whitney U test was applied to determine the difference between the medians of the pre-and post-intervention scores on attitude and practices for psychosocial well-being.

**Results:**

A total of 1040 grade nine students were enrolled. There was a statistically significant increase in median score on attitudes [81.8 (IQR:75.5–85.5) to 82.3(IQR:78.6–87.2] and practices [81.7(IQR: 76.1–85.7) to 83.1(IQR: 79.1–86.9)] in the IA while there was no significant difference in the CA. The proportion of bullied adolescents in the past 30 days reduced significantly from 14.8% (*n* = 38) to 7.9% (*n* = 20) in IA(*p* = .03), whereas there was a slight reduction from 17.1% (*n* = 44) to 11.3% (*n* = 26) in CA (*p* = .17).

**Conclusions:**

The present psychosocial intervention is effective in improving the psychosocial well-being of school adolescents, though long-term effectiveness was not assessed. It is recommended to utilise study findings in deciding to introduce the present intervention to basic and in-service teacher training packages and school curricula with necessary modifications.

**Supplementary Information:**

The online version contains supplementary material available at 10.1186/s12889-023-17023-6.

## Introduction

Adolescents account for 16% of the world’s population, making it essential to cater for adolescent health needs in reaching the targets of Sustainable Development Goals [1]. Adolescents are in a transition period to adulthood where they undergo rapid development, have opportunities for progress, and are vulnerable to developing a multitude of health risks [2]. Mental health issues are identified as a leading factor accounting for disability among adolescents [3]. In global context, mental health problems affect 10–20% of children and adolescents, accounting for 15–30% of disability-adjusted life years lost during the first three decades of life [3]. Over 50% of these conditions appear before the age of 14. Self-harm is the second leading cause of death among 15-to 19-year adolescent females and was the third leading cause of death among males of the same age group [4]. Poor psychosocial well-being leads to increased risk behaviours such as deliberate self-harm, tobacco, alcohol, and illicit substance consumption, and unsafe sexual behaviours [5]. The repercussion of these risky behaviours carries on throughout the life cycle, even for future generations.

In the Sri Lankan context, out of the 20.4 million Sri Lankan population, 16% consists of adolescents [6], of whom 71% are school-going, with most of the non-schoolers between 16 and19 years [7]. The school dropout rate changed very slightly in primary and grades 1–10 over 2016–2019, with respective changes from 0.51% to 0.53%- and 1.37% to 1.74%. The adolescent mortality rate was 41 per 100 000 population in 2019 [8] while the respective global figures ranged from 9 to222 per 100 000 [8], and the South East Asian figures ranged from 32 to 77 per 100 000 [8]. Mortality among young persons in Sri Lankan due to suicide was 8.9 per 100 000 as per the latest available cause-specific mortality data in 2014 [9]. Suicide was the second leading cause of death among both Sri Lankan adolescents and Southeast Asian adolescents in 2019 [10], while in all other regions, it was among first four causes of death [10].

The Global School Health Survey (GSHS) conducted among 13–17-year-old school-going adolescents in Sri Lanka showed that 6.7% had attempted suicide in the past 12 months, while 6.8% had planned to attempt suicide [11]. Nearly 10% seriously considered attempting suicide during the preceding 12 months, and 39% were bullied on one or more days in the past 30 days [11]. Violence among schooling adolescents was identified as a major concern [11, 12]. Further, 8.5% felt lonely within the past one year, and 5.6% had no close friends [11]. Literature shows that loneliness in adolescents has a relationship to depression in adulthood [13]. Of males, 15.3% were currently using tobacco products, while 3.1% of females were current users. The prevalence of current smokers was 3.5%, with 6.2% among males and 0.7% among females [11]. Approximately, 5% of males and 1% of females currently consumed alcohol and 2.7% currently used addictive drugs with 4.2% among males and 1.1% among females [11] The use of tobacco, alcohol, and other substances leads to various psychological and social issues in addition to health concerns [14]. Prevention of the use of addictive substances is essential for the promotion of psychosocial well-being. Over one-third (38.5%) had been bullied on one or more days during the past 30 days [11].

A descriptive cross-sectional study carried out among schooling adolescents aged 13–15 years (*n* = 1770) in the Gampaha district of Sri Lanka, showed that the prevalence of being an overall victim for any violent activity, which was defined as “being physically or psychologically hurt as a result of a specified violent act committed by a child in his or her school, or in another school, or in a tuition class” at least once within the preceding six months was 85.1% [15, 16]. Specified violent acts ranged from name calling/using bad words to hitting as identified in the Sri Lankan Early Teenagers' Violence Inventory (SLETVI) validated for Sri Lanka [17].

Helping Adolescents Thrive (HAT), developed jointly by the WHO and UNICEF, focuses on strengthening programs and policies targeting promoting positive mental health, preventing mental health issues, self-harm, and other risk behaviours in adolescence [18]. Incorporating evidence-based interventions is critical ensuring the psychosocial development of adolescents [4, 7, 19]. Global guidelines on adolescent mental health interventions recommend universal implementation of psychosocial interventions [18, 20]. It further emphasizes the promotion of positive mental health, as well as the prevention and reduction of suicidal behaviour, mental disorders, aggressive, disruptive, and oppositional behaviours, and substance use [20].

Several interventions have been conducted in the promotion of mental health and prevention of psychosocial problems among school going children [21–23]. Fazel et al. (2014) discusses that when the mental health services are integrated with the educational setting, a continuum of care would be delivered promoting the overall health of students, their mental health and their education attainments [23].

Sri Lanka has a well-established school health programme which includes school medical inspections conducted by the Medical Officer of Health (MOH). The health promoting school programme is implemented as a joint activity of the health and education sectors. Important aspects of adolescent health are included in the school curriculum. Teacher counselling services are offered to schools with more than 400 students [7]. Yet, country- specific data reflect the need for well-planned psychosocial interventions targeting the promotion of psychological well-being for school- going adolescents [11, 24].

Therefore, there is a timely need for an evidence-based intervention to improve the psychosocial well-being of adolescents to overcome the psychosocial issues among schooling adolescents in Sri Lanka. Such an intervention could improve the psychosocial well-being of adolescents and minimize risky behaviours and their consequences. This study aimed to assess the effectiveness of an educational intervention delivered through teachers to improve the psychosocial well-being of school-going adolescents in grade nine (aged 14) in the Western Province of Sri Lanka.

## Methods

**Fig. 1:**
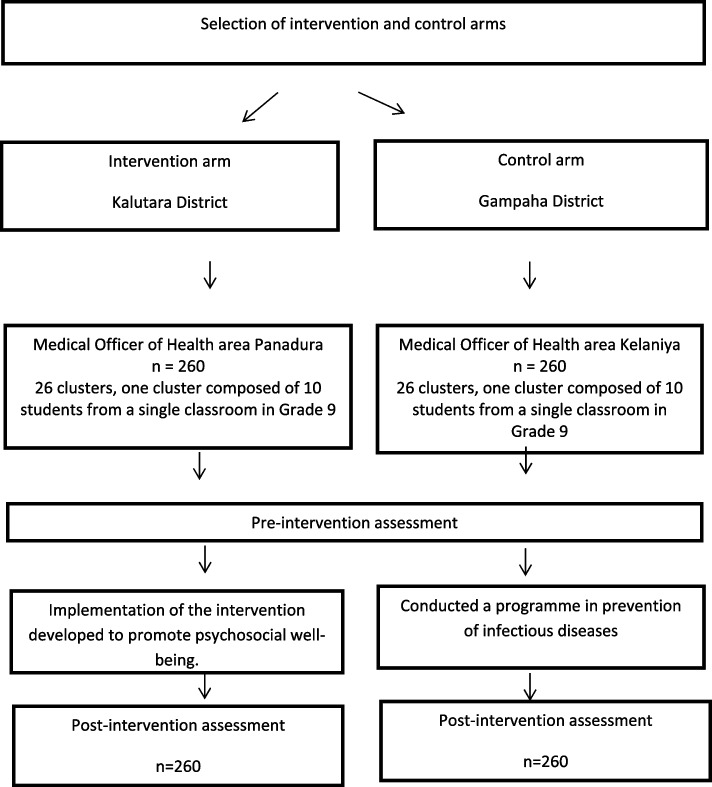
Steps of the study

The steps of the study are outlined in Fig. 1.

### Study design and setting

A school-based quasi-experimental study (Fig. 1) was conducted in 2019. All methods were carried out in accordance with relevant guidelines and regulations.

Sri Lanka has nine provinces, and the Western Province is the most populous province, with nearly one-quarter of the total population. Western province is also home to 13.3% of the total schools in the country, with nearly 1 million of 4.2 million all-age schoolers [25]. Colombo, Kalutara, and Gampaha districts comprise the Western province. Kalutara and Gampaha districts were selected as the intervention area and control area, respectively, as they were socio-demographically similar and situated apart, which would prevent any possible contamination.

For health administrative purposes, each district is divided into Medical Officer of Health (MOH) areas where the population varies from 50,000 to 200,000.

Panadura MOH area was selected randomly from 12 MOH areas in Kalutara district as the intervention area (IA) using the lottery method. From the 16 MOH areas in the Gampaha district, the MOH areas socio-demographically comparable with Panadura MOH area were identified. Socio-demographic comparability was assessed considering the area of residence (urban vs. rural) nationality, and religion. Out of those areas, Kelaniya MOH area was selected randomly as the control area (CA).

### Study participants

Only grade nine was selected as grade 10 and 11 students and teachers were more engaged in preparation for the General Certificate of Education (Ordinary Level) examination; thus, testing a new intervention was not practically feasible due to the tight academic schedule.

For sample size calculation, as there were no previous studies conducted on attitude and practices on psychosocial well-being among schooling adolescents in Sri Lanka, the baseline percentage scores for attitude and practices on psychosocial well-being were taken as 50%. To identify the effectiveness of the intervention, the expected levels of percentage scores of attitudes and practices on psychosocial well-being in the IA at post-intervention assessment were predetermined at 70%. Applying these figures sample size was calculated using the standard sample size calculation formula [26] well-being. The calculated sample size for this quasi-experimental study was 260 for one group. The primary sampling unit was a school, and a cluster was defined as ten students in a grade nine classroom, considering that the minimum number of students per class was 10. Twenty-six clusters each from both IA and CA were selected using the probability-proportional-to-size sampling of the grade nine student population in each school. Simple random sampling was used to select the relevant number of clusters of grade nine classes in each school.

In this process, the government schools, and government-assisted schools with grade nine classes were listed with the number of grade nine students separately for IA and CA. The number of clusters to be taken from each of the school was selected probability-proportionate to the size of the number of grade nine students. Cluster size was considered as 10; thus, 26 clusters were selected by applying the systematic sampling technique. All the government schools in IA were listed with their cumulative population. In both lists, the order of schools listed included girls-only schools, boys-only schools, and mixed schools. The sampling interval was determined by dividing the above figure by the number of clusters (26). A random number was selected within the first sampling interval to determine the starting point. A subsequent cluster was identified by adding a sampling interval to that number. In this manner, the number of clusters to be selected from each school was identified. Clusters in the CA were also identified in a similar manner. From each school selected, grade nine classes were listed. The calculated number of clusters were selected randomly from this list using the lottery method. One class was taken as one cluster. From each of the selected cluster, ten adolescents were selected randomly using the lottery method.

As this was a community intervention, pre- and post-intervention assessments were done among different groups from the same schools to see whether it was effective as a community intervention rather than an individual-based intervention. Individual matching at pre-and post- interventional assessments was not done to ensure same random selection at pre-and post-assessment with in the schools selected for pre-intervention assessment, and results reflecting the effects of the community intervention. No matching of schools by sex was done as there were basically equal numbers of males only, females only, and mixed schools in both IA and CA and the list of schools from each area was prepared in the order of girls only, boys only and mixed schools. Since no data were available regarding mental health and well-being of students in the study area, matching based on baseline mental health and psychological parameters could not be done. As the intervention was a community intervention, which was delivered to all schoolchildren of the selected grade, separate groups in the same schools were selected for the post-intervention assessments from the IA and CA following the same steps mentioned above.

One week before data collection, informed written consent was obtained from parents by sending consent forms home through hand delivery by the student to the parent or guardian and assent was obtained from each student on the day of data collection. All consented to data collection as Sri Lankan students and their parents are very supportive for health-related interventions.

### Design and implementation of the intervention

The educational intervention was developed following an extensive literature review and expert consultations based on a need assessment carried out by the School Health Unit of the Family Health Bureau, Ministry of Health, the national level focal point for school health. There were health promotion materials developed and circulated among adolescents and their parents on mental health promotion, parenting, and life skills by the School Health Unit. Yet, there was a lack of an organised intervention addressing psychosocial well-being holistically, targeting the adolescents’ psychosocial development and mental health issues. Thus, an educational intervention consisting of an intervention delivered through trained health teachers targeted at improving psychosocial well-being of school-going adolescents was developed.

This package consisted of a teachers’ guide, trainer manual, and activities to be delivered to students by health teachers. These teaching and learning materials and information and communication materials were developed by a multi-disciplinary panel of experts representing public health, paediatrics, child psychiatry, clinical psychologists, and the education sector. Inputs and feedback from experts in all relevant fields, including health teachers and students were obtained.

Training of trainer programme was conducted for district- level supervisory public health staff in the IA. They conducted two-day training programmes for health teachers in all the government and government-assisted schools in the area. Training programme for teachers addressed many important aspects including the psychosocial development of adolescents, capacity building of teachers on prevention, early identification and referral of adolescents related to mental health issues, prevention of violence, bullying, tobacco, alcohol, and substance abuse, health issues of vulnerable groups, mindfulness, development of ten key life skills and safe use of information technology. Further, it focused on mental health promotion in schools, teachers’ role in handling difficult children, and multi sector approach improving the psychosocial health of schoolchildren. Teachers developed their skills of delivering the package through activity-based learning, which was like how they are intended to deliver it to the students. This approach was applied to develop skills and build confidence in teachers, in addition to improving their knowledge. The trainer package included a pre-test and a post-test to ensure that teachers acquired the necessary knowledge and competencies.

The psychosocial intervention package was delivered to the students through a package of classroom activities. Teachers teaching health subject at schools were selected as they were technically sound on health and well-being. Teaching instructors of the zonal education office, who were trained on this package by district-level public health staff, monitored the delivery of the intervention package by health teachers to ensure a standard way of delivery.

The psychosocial health promotion package implemented by teachers consisted of nine sessions with 24 activities, based on the different modules of the psychosocial health promotion guide (Supplement 1). Based on the availability of time, teachers were instructed to decide whether they would complete a combination of many activities or one or two activities on a given day. These activities aimed to adolescents’ skills and were delivered as short activity-based sessions of 20 min during normal teaching periods by the health teacher. Case scenario-based discussions, games, role-plays, and group work with brainstorming sessions involving 8–10 students per session were the common methods used to deliver the package to the students (Supplement 1-Pages 60–80). Group presentations, where relevant, allowed the teachers to understand whether the students had achieved the expected learning outcomes. Adolescents with mental health issues were confidentially referred to the medical officer of health in the area. None of the details of such children were divulged to other teaching staff.

The educational intervention was carried out in all schools in the Kalutara district; however, only the selected schools in Panadura MOH area (IA) were considered from the interventional district for the assessment. In the control district, district-level training programmes and teacher training programmes were conducted on the prevention of infectious diseases.

### Pre- and post-intervention assessment

Pre-intervention assessment was conducted one month before the intervention by four pre-intern doctors using a pre-tested interviewer-administered questionnaire (IAQ), which consisted of socio-demographic data, case scenarios, attitudes, and practices on psychosocial well-being, as well as risky behaviours hindering psychosocial well-being such as bullying, suicidal ideas, addictive behaviours, and alcohol and tobacco use. Case scenarios were included to assess students’ attitudes towards applying their knowledge of psychosocial health to make decisions in confrontation with such situations. Attitudes were measured on a five-point Likert scale. The final questionnaire was pre-tested among 25 grade nine students at a school in Colombo district which was not within study districts. Necessary changes were done to improve comprehension and user-friendliness based on the pre-test findings.

Intervention was conducted over three months, and just after the completion of three months, post- intervention assessment was done by the same data collectors using the same IAQ used in the pre-intervention assessment. After the completion of the three months of implementation, qualitative assessments on the success of the intervention were conducted as focus group discussions among teachers and students in IA, and findings reflected the importance and success of the intervention.

### Data analysis

Data entry and analysis were done using SPSS Version 29.1. Descriptive statistics were used to describe the study population. Scoring systems were developed to assess the attitudes and practices on psychosocial well-being of adolescents. Attitudes were assessed using 18 items with a Likert scale ranging from 0 to 4. The most favourable response per item for psychosocial well-being was scored as 4, and the most unfavourable response was scored as 0. Eighteen items covered attitude on happiness, extracurricular activities, future goals, family relationships, life skills, mindfulness, problem-solving, having friends, respecting others, protectiveness, violence, bullying, tobacco use, alcohol use, and risky instances in using social media. Hence, the scores ranged from 0 to 72. Reliability assessment of the scale showed that Cronbach's coefficient alpha for the scale on attitude was 0.8.

Practices were assessed using 0 and 1, and the scores ranged from 0 to 22. Items on practices on psychosocial well-being covered happiness, having friends, mindfulness, participation in social events, life skills, problem-solving, loneliness, stress, anger management, protectiveness, relationships, violence, bullying, tobacco smoking, alcohol use, and substance use. Cronbach's coefficient alpha for the scale of practices for psychosocial well-being was also 0.8.

These scores were converted into percentage scores and presented with median values with respective interquartile ranges (IQR) as all scores did not follow a normal distribution. The cut-off value for satisfactory levels of attitude and practices on psychosocial well-being was predetermined as over 70%. Within-group and between-group comparisons of the categories based on the level of percentage scores were conducted using the Chi-Square test and Fisher’s exact test as applicable at *p* < 0.05.

Further, Mann–Whitney U test was used to assess the statistical significance of the differences in the percentage scores in both pre-and post-intervention assessments and between-group comparisons. Between-group comparisons for categorical variables were conducted using Chi-Square test and Fisher’s exact test as applicable *p* < 0.05 was considered statistically significant in all analysis.

Ethical clearance was obtained from the Ethics Review Committee of the Faculty of Medicine, University of Colombo (number EC-19–069) before the commencement of the study. Informed written consent from the parents were obtained by sending consent forms home one week before data collection, as the literacy rate of the population in the area was 96% [6], and assent from the participants was obtained before data collection. All the parents consented to the study and all selected students provided their assent.

## Results

Pre- and post-interventions were completed by 1040 respondents, with 260 for each arm. The mean age of the participants at the pre-intervention was 14.1 (SD = 0.32) years in IA and 14.1 (SD = 0.38) years in CA (*p* > 0.05). Cronbach’s coefficient alpha for the scales on both attitude and practices for psychosocial well-being was 0.8.

Sociodemographic characteristics are as indicated in Table 1.

**Table 1:** Comparison of socio-demographic characteristics of study participants in the intervention (IA) and control (CA) areas at pre-intervention and post-intervention

Characteristic	Pre-Intervention					Post-Intervention				
	**Intervention Group (*N* = 260)**		**Control Group (*N* = 260)**		**Statistical Significance**	**Intervention Group (*N* = 260)**		**Control Group (*N* = 260)**		**Statistical Significance**
**Sex**					χ2 = 3.48 df = 1 *p* = .06					χ2 = 4.3 df = 1 *p* = .04
Male	100	38.6%	121	46.75		71	27.3%	92	35.8%	
Female	159	61.4%	138	53.3%		189	72.7%	165	64.2%	
	259		259			260		257		
**Ethnicity**					χ2 = 0.04 df = 3 *p* = .84					χ2 = 1.48 df = 1 *p* = .23
Sinhala	246	94.6%	247	95.0%		243	93.8%	249	96.1%	
Other	14	5.4%	13	5.0%		16	6.2%	10	3.9%	
	260		260			259		259		
**Religion**					χ2 = 0.51 df = 1 *p* = .48					χ2 = 6.49 df = 1 ***p* = .01**
Buddhism	235	90.4%	230	88.5%		240	92.7%	222	85.7%	
Other	25	9.6%	30	11.5%		19	7.3%	37	14.3%	
	260		260			259		259		

*X*^*2*^ Chi square test

### Overall scores on attitudes on psychosocial well-being

Tables 2 and 3 provides a summary of within, and between-group comparisons of attitudes and practices on psychosocial well-being among grade nine students in intervention and control groups.

**Table 2:** Within and between group comparison of percentage scores for attitudes and practices on psychosocial well-being among grade nine students in in intervention and control groups

Component on Psychosocial Well-being	Group	Pre-intervention percentage score Median (IQR)	Post-intervention percentage score Median (IQR)	Within-group Comparison Statistical Significance	Between-group Comparison Statistical Significance	
					**Pre-intervention**	**Post-intervention**
**Attitude**	Intervention Group	81.8(75.5–85.5)	82.3(78.6–87.2)	***p* = .004**	*p* = .939	***p* = .015**
	Control Group	81.8(77.7–85.0)	81.4(75.9–87.2)	*p* = .735		
**Practices**	Intervention Group	81.7(76.1–85.7)	83.1(79.1–86.9)	***p* = .002**	*p* = .810	***p* = .036**
	Control Group	81.7(77.8–84.8)	82.2(76.9–86.1)	*p* = .419		
**Total**	Intervention Group	81.4(76.0–85.5)	82.5(78.9–85.9)	***p* = .005**	*p* = .986	*p* = .106
	Control Group	81.4(77.3–84.4)	81.8(76.4–85.9)	*p* = .429

**Table 3:** Within and between group comparison of levels of attitudes and practices on psychosocial well-being among grade nine students in in intervention and control groups

Component on Psychosocial Well-being	Group	Stage	Level of Percentage Score		Within-group Comparison Statistical Significance	Between Group Statistical Significance	
			**Low (≤ 70%)**	**Satisfactory (> 70%)**		**Pre-Intervention**	**Post-Intervention**
**Attitude**	Intervention	Pre	23(8.8%)	237(91.2%)	*p* < .001*	*p* = .520**	*p* < .001*
		Post	4(1.5%)	256(98.5%)			
	Control	Pre	19(7.3%)	241(92.7%)	*p* = .169**		
		Post	28(10.8%)	232(89.2%)			
	Intervention	Pre	36(13.8%)	224(86.2%)	*p* < .001*	*p* = .802**	*p* < .001*
**Practice**		Post	2(0.8%)	258(99.2%)			
	Control	Pre	38(14.6%)	222(85.4%)	*p* < .001**		
		Post	85(32.7%	175(67.3%)			
	Intervention	Pre	43(16.5%)	217(83.5%)	*p* < .001*	*P* = .727**	*p* < .001*
**Total**		Post	3(1.2%)	257(98.8%)			
	Control	Pre	46(17.7%)	214(82.3%)	*p* < .001*		
		Post	97(37.3%)	163(62.7%)			

^*^Fisher’s exact test

^**^Chi-Square test

### Between-group analysis

At pre-intervention assessment, the median percentage score of the IA and CA both were 81.8 with respective IQRs of 75.5–85.5 and 77.7–85.0 (*p* = 94) (Table 2). However, at post-intervention assessment, the median percentage score was significantly higher in IA (82.3; IQR: 78.6–87.2) than in CA (81.4; IQR: 80.0–85.9) [*p* = 0.02].

There was no significant difference in those having a satisfactory level of attitude (with a score > 70%) between two areas at pre-intervention assessment (*p* > 0.05), while there was a statistically significant difference between IA (*n* = 256,98.5%) and CA (*n* = 232,89.2%) at post- intervention assessment (*p* < 0.001) (Table 3).

### Within-group analysis

The median percentage score on psychosocial well-being was significantly higher in IA at post-intervention (82.3; IQR: 78.6–87.2) (Table 2) compared to pre-intervention level (81.8; IQR:75.5–85.5) [*p* = 0.004].

Even though the median percentage scores at post-intervention (81.4; IQR:75.9–86.3) were slightly lower than the pre-intervention value (81.8; IQR:77.7–85.0) in CA, this difference was not statistically significant(*p* = 0.74) (Table 2).

Students with a satisfactory level of attitude (with a score > 70%) increased significantly from 23 (8.8%) to 237 (91.2%) from pre- intervention to post- intervention in the IA (*p* < 0.001), while there was no statistically significant increase observed in the CA (*p* = 0.17) (Table 3).

### Overall scores on practices on psychosocial well-being

#### Between-group analysis

The median percentage score of the IA and CA both were 81.7 at pre-intervention, with respective IQRs of 76.1–85.6 and 77.8–84.8(*p* = 0.81). The median percentage score was significantly higher in IA (83.0; IQR: 79.1–86.9) than in CA (82.2 IQR: 76.9–86.1) [*p* = 0.04] at post-intervention assessment (Table 2).

There was no statistically significant difference between those having satisfactory level of scores for practices at pre-intervention between two groups (*p* = 0.80). However, those with satisfactory level of practices were significantly higher in the IA (*n* = 258,99.2%) compared to the CA (*n* = 232,89.2%) at post-intervention (*p* < 0.001) (Table 3).

#### Within-group analysis

The median percentage score on psychosocial well-being was significantly higher in IA at post-intervention (83.1; IQR: 79.1–86.9) compared to pre-intervention (81.7; IQR: 76.1–85.7) [*p* = 0.002]. The median percentage score at post-intervention (82.2; IQR:76.9–86.1) was slightly higher than the pre-intervention value (81.7; IQR = 77.8–84.8) in CA, though this difference was not statistically significant (*p* = 0.42) (Table 2).

Students with a satisfactory level of practices increased significantly from 224(86.2%) to 258(99.2%) from pre- intervention to post- intervention in the IA (*p* < 0.001), while there was a statistically significant decrease from 222(85.4%) to 175(67.3%) in the CA (*p* < 0.001) (Table 3).

Table 4 summarises the within-group comparisons of changes in practices for psychosocial well-being over time from pre-intervention to post-intervention. The number of students who were never bullied in the past 30 days increased from 219 (85.2%) to 234(92.1%) in IA (*p* = 0.01) and from 214 (82.9%) to 227 (89.7%) in CA (*p* = 0.03). The number of students who never felt lonely within the past 30 days increased significantly from 75 (29%) to 96(37.5%) in the IA (*p* = 0.04), while the number decreased from 91 (35.1%) to 88 (35.1%) in the CA (*p* = 0.96).

**Table 4:** Within area comparison of the practices on psychosocial well-being including problem solving among grade nine students in in intervention (IA) and control (CA) areas

Variable	Intervention Area					Control Area				
	**Pre-Intervention**		**Post Intervention**		**Significance**	**Pre- Intervention**		**Post intervention**		**Significance**
	**No**	**(%)**	**No**	**(%)**		**No**	**(%)**	**No**	**(%)**	
**Number of days been bullied during past 30 days**					χ2 = 6.07 df = 1 ***p* = .02**					χ2 = 4.96 df = 1 ***p* = .03**
0 times	219	85.2%	234	92.1%		214	82.9%	227	89.7%	
1 or more times	38	14.8%	20	7.9%		44	17.1%	26	10.3%	
	257		254			258		253		
**Frequency of been felt lonely during past 30 days**					χ2 = 8.04 df = 3 ***p* = .04**					χ2 = 0.29 df = 3 *p* = .96
Never	75	29.0%	96	37.5%		91	35.1%	88	35.1%	
Rarely	108	41.7%	78	30.5%		90	34.7%	91	36.35	
Some times	57	22.0%	65	25.4%		59	22.8%	55	21.9%	
Most of the time	19	7.3%	17	6.6%		19	7.3%	16	6.4%	
	259		256			259		251		
**Frequency of consuming alcohol in past 30 days**					Fisher’s Exact Test *p* = 1.00					Fisher’s Exact Test *p* = .72
Never	256	99.6%	257	99.6%		253	98.8%	243	98.4%	
Once or more	1	0.4%	1	0.4%		3	1.2%	4	1.6%	
	257		258			256		247		
**Frequency of consumption of illicit drugs in past 30 days**					Fisher’s Exact Test *p* = 1.00					Fisher’s Exact Test *p* = .50
Never	258	99.2%	256	99.2%		258	99.2%	250	100.0%	
Once or more	2	0.8%	2	0.8%		2	0.8%	0	0.0%	
	260		258			260		250		
**Frequency of practicing mindfulness in past 30 days**					χ2 = 0.91 df = 2 *p* = .64					χ2 = 2.01 df = 2 *p* = .37
Not at all	69	27.0%	65	25.1%		64	24.9%	74	30.6%	
Once or twice	77	30.1%	88	34.0%		76	29.6%	66	27.3%	
Most of the days	110	43.0%	106	40.9%		117	45.5%	102	42.1%	
	256		259			257		242		
**Variable**	**Intervention Area**					**Control Area**				
	**Pre-Intervention**		**Post-Intervention**		**Significance**	**Pre-Intervention**		**Post-Intervention**		**Significance**
	**No**	**(%)**	**No**	**(%)**		**No**	**(%)**	**No**	**(%)**	
**You feel unable to do exam next week due to attendance to national sports. What will you do?**					χ2 = 2.27 df = 1 *p* = .13					χ2 = 1.85 df = 1 *p* = .17
Plan and use the available time efficiently or get the help of parents and teachers and do the exam	197	76.7%	206	82.1%		214	83.6%	190	78.8%	
Other responses	60	23.3%	45	17.9%		42	16.4%	51	21.2%	
	257		251			256		241		
**Got attracted to someone of opposite sex. You feel that parents would not be happy of heaving a relationship with that person. What will you do?**					χ2 = 0.45 df = 1 *p* = .50					χ2 = 5.68 df = 1 ***p* = .02**
Seek advice from the parents or Ignore it thinking that it’s not the correct time	238	95.2%	241	96.4%		246	96.5%	211	91.3%	
Other responses	12	4.8%	9	3.6%		9	3.5%	20	8.7%	
	250		250			255		231		
**Friends are forcing you to take alcohol. You do not want. What would you do?**					χ2 = 0.04 df = 1 *p* = .84					χ2 = 0.10 df = 1 *p* = .76
Refrain from drinking	247	95.0%	246	94.6%		243	94.6%	231	93.9%	
Other responses	13	5.0%	14	5.4%		14	5.4%	15	6.1%	
	260		260			257		246		
**New batch of students came. Your friends want to make fun at them. What will you do?**					χ2 = 1.45 df = 1 *p* = .23					χ2 = 0.09 df = 1 *p* = .76
Stop your friends and explain the importance of friendliness or refrain from ragging	216	84.4%	228	88.0%		216	83.7%	216	84.7%	
Other responses	40	15.6%	31	12.0%		42	16.3%	39	15.3%	
**Your best friend offers you a cigarette. He/she says that is something good and that you should try it. What would you do?**					Fisher’s Exact Test					χ2 = 0.01 df = 1
Do not accept the cigarette or Ignore the request	253	98.1%	256	99.2%	*p* = .45	250	97.3%	240	97.1%	*p* = .94
Other responses	5	1.9%	2	0.8%		7	2.7%	7	2.9%	
	258		258			257		247		

In IA, the number of students who had not taken alcohol within the past 30 days remained high and the same at 99.6% (*p* = 1.0), while that figure remained high and the same in CA at 98.4% (*p* = 0.72). Similarly, the number of students who had not taken drugs within the past 30 days remained high (over 99.0%) in both areas. In IA, the majority practiced mindfulness on most days over the past 30 days, with respective figures of 110(43.0%) at pre-intervention and 106(40.9%) at post-intervention assessments (*p* = 0.82). In CA also, these figures were high though the number slightly reduced from 117(45.7%) to 102(42.2%) [*p* = 0.37].

The number of students who claimed that they were taught anger management increased significantly from 150(57.9%) to 165(64.5%) in IA (*p* = 0.12), while the number remained basically the same with respective values of 171(66.3%) and 170(66.5%) in CA (*p* = 0.42). The number of students who received activity-based life skill teaching increased from 189(74.1%) to 210(81.7%) in IA (*p* = 0.04) while the number slightly reduced from 222(86.0%) to207(83.1%) in CA (*p* = 0.37).

For the question related to what students would do when they feel that they are unable to face examinations (as they did not have enough time for the preparation because of attending national sports events), those who responded with the most accurate decision of ‘planning and using the available time efficiently and facing the examination or getting the help of parents and teachers increased from 197 (76.7%) to 206 (82.1%) in IA (*p* = 0.13). Those who provided the above accurate decision in CA decreased from 214 (83.6%) to 190 (78.8%). This decrease was not statistically significant (*p* = 0.17).

In relation to the issue of being attracted to the opposite sex despite parental disapproval, the number of students with favourable responses that they would seek advice from the parents or ignore it thinking that it would not be the correct time, increased from 238 (95.2%) to 241 (96.4%) in IA (*p* = 0.50). In CA, the number of participants with above-favourable responses reduced significantly from 246 (96.5%) to 211 (91.3%) [*p* = 0.02].

Regarding decision-making on alcohol consumption, the number of students who provided responses related to rejecting alcohol despite peer pressure remained high in both areas, with figures of 247(95.0%) and 246(94.6%) in IA (*p* = 0.84), and figures of 243 (94.6%) and 231 (93.9%) in CA (*p* = 0.76).

With regard to decision-making on ragging newcomers to the school, the number of students who responded as not supporting ragging by expressing that they would stop their friends and explain the importance of friendliness or refrain from ragging increased from 216(84.4%) to 228(88.0%) in IA (*p* = 0.23), while that figure remained at 216(84%-85%) in CA(*p* = 0.76).

About the decision-making on cigarette smoking when their best friend offers a cigarette saying that it is something good, the number of students who responded that they would not resort to smoking despite peer pressure by expressing that they would not accept the cigarette or ignore the request slightly increased from 253(98.1%) to 256(99.2%) in IA (*p* = 0.45) and changed from 250(97.3%) to 240(97.1%) in CA(*p* = 0.94).

## Discussion

This quasi-experimental study conducted among 1040 school-going adolescents of grade nine in the Western Province to assess the effectiveness of a psychosocial health promotional intervention showed a statistically significant increase in the median scores for attitudes (*p* = 0.004) and practices (*p* = 0.002) related to psychosocial well-being in the IA, while there was no such statistically significant increase in attitudes and practices in the CA (*p* > 0.05). This reflects that the intervention was effective in improving psychosocial well-being among adolescents. It is noteworthy to mention that this significant improvement has been observed only after a brief intervention period of three months; thus, given that the intervention is supposed to be carried out for a longer period incorporated within the school curriculum, it could be expected to achieve substantial, sustained improvement in the psychosocial well-being of school adolescents in the future.

School-based interventions to promote adolescent health have been shown to be effective throughout the globe. A systematic review conducted by Xu et al. (2019) on school-based interventions to promote adolescent health in low- and middle-income countries of the WHO Western Pacific Region showed that school-based interventions are effective in increasing knowledge, attitude, and practices on tobacco use, suicide, obesity, and sexual and reproductive health [22, 27–29]. The studies included in it mainly focused on adolescents. Comparable to the present intervention, some of the studies included in the review had educational interventions targeted at the prevention of tobacco use. Of them, only one study focused on life skills. However, the small number of studies and small sample sizes of some studies included in the assessment were some of the limitations of this review [30].

In the present study, the percentage of those who never felt lonely within the past 30 days increased significantly in the IA (*p* = 0.04), while the corresponding value decreased in the CA (*p* = 0.96). Those who were never bullied in the past 30 days increased significantly in the IA (*p* = 0.01), while there was only a slight increase in the CA (*p* = 0.02). Having conducted the post-intervention assessment following a relatively short period of three months could be a possible reason for not having higher improvement on these outcomes. These results are comparable with the findings of a recent meta-analysis conducted using 100 eligible studies, which showed that anti-bullying programmes significantly reduced bullying perpetration (OR = 1.3) and bullying victimization (OR = 1.2) [31]. It suggested that anti-bullying programmes reduced perpetration by 19–20% and bullying victimization by 15–16% within school. However, there was a considerable variation in effect sizes across these studies, with a significant heterogeneity between studies for both bullying perpetration and bullying victimization outcomes [31].

In relation to alcohol and illicit drug consumption, those who never consumed in both areas were over 98% in both pre-and post-intervention assessments. Consequently, eliciting improvement over time was not reflected in both areas. Marked improvement on these aspects were not expected as alcohol and illicit drug use at baseline level were low. Yet, inclusion of interventions targeting life skill-based decision-making of non-consumption of alcohol and illicit drug consumption in intervention was vital as adolescent behaviour tends to change by peer influence, indirect advertising through social media, etc., and it was essential to sustain good practices [32, 33]. Further, as the data were based on self-reporting by adolescents, there might have been a certain under-reporting of these aspects due to the possible perception of cultural norms in the country being against such practices. In the IA, the majority practiced mindfulness on most days over the past 30 days, with respective figures of 110 (43.0%) at the pre-intervention and 106 (40.9%) at post-intervention stage (*p* = 0.82). In the CA also, these figures were high though it was slightly reduced from 117 (45.7%) to 102(42.2%) [*p* = 0.37]. The observed slightly higher values at pre-intervention might be due to a separate mindfulness programme carried out in schools in both districts named as “Sati Pasala” just before the intervention. Another fact that might have affected not improving mindfulness practices over time, is that the post-intervention assessment was done closer to the year-end examination. No other specific programmes were conducted in either IA or CA within the period of the present intervention.

For the question of whether they were taught how to control anger, those who expressed ‘yes’ increased from 150 (57.9%) to 165 (64.5%) in IA (*p* = 0.12). In CA, the respective figures remained the same, with pre-intervention value of 171 (66.3) and post-intervention figure of 170 (66.5%) [*p* = 0.42]. Regarding the question of whether they were trained on activity-based life skills at school, the number trained increased significantly from IA over time (*p* = 0.04) while that figure in CA slightly decreased [*p* = 0.39]. This finding reflects the successful implementation of the present intervention. Improvements on these aspects were shown in a school-based intervention conducted targeting the life skill development of adolescents through teachers among one hundred fifty-nine adolescents (mean age ± SD = 15.7 ± 1.4 years) randomized as an intervention group (*n* = 86) or control group (*n* = 73) in Augusta, Georgia across two years. It showed that anger and anxiety scores decreased in the intervention group across the six-month follow-up period compared to the control group (*p* < 0 0.05). The intervention group engaged in twelve 50 min life skills training sessions conducted by teachers at school [21].

In the present study, the problem-solving skills of participants were assessed regarding their decision-making capacity in different case-based scenarios, which very well reflects their ability of decision making in psychosocial issues. Scope of these case-based scenarios covered all the important aspects of psychosocial well-being. As reflected in Table 4 problem-solving skills showed an increase in IA, while there was no such increase in CA. This is supported by the findings of practices, where the median percentage score of practices on psychosocial well-being increased significantly in IA from 81.7 (IQR:76.1–85.7) to 83.1 (79.1–86.9) [*p* = 0.002] while that in CA increased only slightly from 81.7 (77.8–84.8) to 82.2 (76.9–86.1) [*p* = 0.42]. Even though the overall median score increased slightly, there was a marked improvement in students with low percentage scores. These students with low scores are in danger of developing psychosocial issues. Therefore, this intervention has a good impact on them. Qualitative assessment among health teachers and students who underwent this intervention also reflected the importance and improvements in psychosocial well-being among adolescents.

One of the main strengths of the present study was the selection of the IA and CA, which were socio-demographically similar and situated apart, which prevented any possible contamination. As this was a group intervention, pre- and post-assessments were done in separate groups within the area. This prevented repeated application of the same study instruments among the same study participants, which was likely to lead to an improvement in response to repeated measurements.

### Limitations

In the present study, matching was not done based on school characteristics. One of the main limitations was that no data were available on the mental health and well-being of students in the study area for such matching.

Another limitation of the present study was the short follow-up period of three months. School closure due to the COVID-19 pandemic prevented reassessment at six months and one year, which would have provided more data relevant to the sustainability and output of the intervention over a longer period. Further, as this intervention was developed before the COVID-19 pandemic started, the present intervention did not cater for improving psychosocial well-being during the home-bound period. For such instances, modifications are needed to be done to the intervention to help adolescents’ psychosocial well-being through virtual platforms by teachers. The timing of the pre-and post-assessment was also not optimal, considering the academic activities conducted during the assessments. At the pre-intervention assessment students were more relaxed as the academic term had just started, while at the post-intervention assessment, they were more focused on the examination. This could have resulted in an underestimation of the effectiveness of the intervention. However, such an under-estimation is practically expected in similar studies.

As this was a community intervention, pre- and post-intervention assessments were done among different groups from the same schools to see whether it was effective as a community intervention rather than an individual-based intervention. However, the systematic differences among participants by sex between two areas is a main limitation of the study. The generalizability of the findings to the entire country is limited as the intervention was carried out only in one district. However, Western Province is the province with highest population in the country (28.7%) [6].

### Public health implications

The present study shows the effectiveness of the educational intervention tested to promote the psychosocial well-being of school-going adolescents. Considering the existing status of psychosocial health and well-being of students in school settings, even the small, significant improvement observed over only three months is very important. As mentioned above, if the intervention was carried out for a longer period incorporated within the school curriculum, it could be expected to achieve substantial, sustained improvement in the psychosocial well-being of school adolescents in the future. Further, there were no ongoing interventions specifically targeting psychosocial well-being of the school going adolescents in Sri Lanka by the time this intervention was tested. Yet, long- term and periodic assessments following the implementation of the intervention and comparison with other psychosocial interventions coming up in the region are needed, as there are no other psychosocial interventions still tested in Sri Lanka.

Considering the logistic feasibility, we further recommend including the training manual developed in the study during the basic training of the teachers before their in-service training in such future interventions.

### Supplementary Information

**Additional file 1.**

## Data Availability

The datasets generated and analysed during the present study are not publicly available due to the need of ensuring confidentiality but are available from the corresponding author on reasonable request.

## Abbreviations

AA-HA: Accelerated Action for the Health of Adolescents
e-IMMR: E-Indoor Morbidity and Mortality Register
GSHS: Global School Health Survey
IQR: Interquartile Range
IA: Intervention Area
CA: Control Area
MOH: Medical Officer of Health
PI: Principal Investigator
RDHS: Regional Director of Health Services
SAQ: Self-Administrated Questionnaire
SD: Standard Deviation
SMI: School Medical Inspection
UNFPA: United Nations Population Fund
UNICEF: United Nations International Children's Emergency Fund
WHO: World Health Organization
