# Supplementary material for: Effectiveness of an educational intervention to promote psychosocial well-being of school-going adolescents in Sri Lanka
Source: BMC Public Health. 2023 Nov 7;23:2185. doi: 10.1186/s12889-023-17023-6 (PMC10631107; doi:10.1186/s12889-023-17023-6)

Psycho-Social Health Promotion of School Children

Mannual for Trainers

Family Health Bureau

Ministry of Education

Printed and published by School Health Unit of
Family Health Bureau of Ministry of Health, Nutrition and Indigenous Medicine in 2019

Publication Date: 2019. 06. 30

School Health Unit
Family Health Bureau
Ministry of Health, Nutrition and Indigenous Medicine
Sri Lanka

Tel: 0112692746
Fax: 0112692746
E mail: Sh_fhb@yahoo.com, schoolfhb@gmail.com
Web site: www.fhb.health.gov.lk

Panel of Advisors

Dr. Ayesha Lokubalasooriya

MSc, MD in Community Medicine
Consultant Community Physician
National Programme Officer–School Health
School Health Unit
Family Health Bureau

Dr. Chiranthika Vithana

MSc, MD in Community Medicine
Consultant Community physician
Adolescent and Youth Health
Family Health Bureau

Prof. Hemamali Perera

MD (Child and Adolescent Psychiatry),FRCPsych(UK)
Senior Lecturer, University of Colombo
Former Consultant Child and Adolescent Psychiatrist
Lady Ridgeway Hospital

Prof. Deepthi Samarage,

MD in Paeiatrics, MRCP(UK)
Professor of Peadiatrics of South Asian Institute of Technology and Applied Medicine (SAITAM)
Former Professor of Peadiatrics, University of Sri Jayawardhenapura

Dr. Swarna Wijethunga

MD in Psychiatry
Consultant Child & Adolescent Psychiatrist
Lady Ridgeway Hospital

Dr. Jayamal De Silva

MD in Psychiatry
Consultant Psychiatrist
& Senior Lecturer in Psychiatry,
University of Sri Jayawardhenapura
Member, Expert Committee on Tobacco and Alcohol, SLMA

Dr. Manjula Danansooriya

MSc, MD in Community Medicine
Consultant Community Physician
World Health Organization

Dr. Monika Wijerathne

MBBS, MSc, MD in Community Medicine
Consultant Community Physician
Office of the Provincial Director of Health Services, Western Province

Dr. Nuwan Wickramasinghe

MBBS, Dip (OH&S), MSc (App. Epid.), MSc (Com. Med.), MD in Community Medicine, FRSPH
Consultant Community Physician
Senior Lecturer in Community Medicine
Medical Faculty
University of Rajarata

Dr. Sameera Senanayake

MSc, MD in Community Medicine
Senior Registrar in Community Medicine
School Health Unit
Family Health Bureau

Dr. Yasara Samarakoon

MBBS, MSc, MD in Community Medicine
Senior Registrar in Community Medicine
Medical Faculty, Colombo
Former AMOH Biyagama

Dr. Chinthika Liyanage

MBBS, Dip: in Child Health
Medical Officer
School Health Unit
Family Health Bureau

Mr. Anura Abewickrama,

BA, M Phil, B. Ed, M. Ed, Dip in Edu., Dip in Physical Edu. , Dip in Edu. Management, Dip in Teaching, SLEAS (III)
Assistant Director in Education,
Physical Education Unit
Ministry of Education

Authours

Dr. Dushyanthi M.S. Jayawardene

ATCL, MBBS, PGCert (Med Ed), CTHE (Col), SEDA (UK), MSc (Comm Med), MD (CommMed)
Consultant Community Physician,
Senior Lecturer, Department of Community Medicine, Faculty of Medicine,
University of Colombo.

Dr.Himali Herath

MBBS, MSc, Md, (Community Medicine)
Senior Registrar - Community Medicine
School Health Unit
Family Health Bureau

Editors

Dr. I. O. K. K. Nanayakkara
MBBS, MSc (Community Medicine)
Medical Officer,
School Health Unit, Family Health Bureau

Dr. Himali Herath
MBBS, MSc, MD, (Community Medicine)
Senior Registrar - Community Medicine
School Health Unit, Family Health Bureau

Mr. G. Dhammika Kodithuwakku
Senior Lecturer
National Programme Officer,
Health and Physical Education
National Institute of Education

Dr. Kanchana Mahagamage
MBBS
Medical Officer, School Health Unit
Family Health Bureau

Translators Sinhala

Dr. I.O.K. K. Nanayakkara
MBBS, MSc (Community Medicine)
Medical Officer,
School Health Unit, Family Health Bureau

Dr. Kanchana Mahagamage
MBBS
Medical Officer, School Health Unit
Family Health Bureau

Tamil

Dr. G. Pragasan
MBBS, MSc (Community Medicine)
Registrar - Community Medicine,
School Health Unit, Family Health Bureau

Mrs. Kathireson Nesamalar
Public Management Assistant
Health Promo on Bureau

Mr. M. H. A. Razeem
Development Officer
Derector Nursing & Medical Services, Ministry of Health

Computer Type Setting & Page makeup

Mr. R.P. Nuwan Sameera
Public Health Inspector
School Health Unit
Family Health Bureau

Photographs

Mr. R.P. Nuwan Sameera
Public Health Inspector
School Health Unit

Resources

Mr. Anil Karunathilake - Development Officer, School Health Unit, Family Health Bureau

Miss Darshani Kumari - Development Officer, Family Health Bureau
Mr. Sudesh Kumarasinghe

Contents

Session	Page No
**Session 1**<br>Psychosocial Development of Adolescence	01
**Session 2**<br>Adolescent Brain Development	04
**Session 3**<br>Risk factors and protective factors for Adolescent mental health disorders	07
**Session 4**<br>Stress and Educational Problems in Adolescence	11
**Session 5**<br>Mental Health Problems of Adolescents and Children	14
**Session 6**<br>Mental Health Aspects of Sexual &<br>Reproductive Health Problems in Adolescence	16
**Session 7**<br>Impacts of Bullying at School	19
**Session 8**<br>Violence Its Consequences in adolescence	23
**Session 9**<br>Effects of Child Abuse on Childhood and Adolescence	27
**Session 10**<br>Chronic Physical illness-the Effect on Mental Health	31
**Session 11**<br>Impacts of Information Technology and Media on Adolescence	33
**Session 12**<br>Health Issues of Vulnerable Adolescent Groups	35
**Session 13**<br>Counseling of school children for Substance abuse,<br>a practical approach for teachers	38
**Session 14**<br>Teachers ' Role in Handling Difficult Children	41

Session 15
Mental Health Promotion at School 43

Session 16
Life Skills – A Way Out for Mental Health Problems 46

Session 17
Mindfulness – A recognized intervention for Psychosocial Developement 49

Session 18
Multi Sectoral Approach to Improve psychosocial Health of Children 52

Annextures

Annex I. Taking a History of a Presenting Problem/Concern from an Adolescent 55

Annex II. Psycho Social Health Promotion among School Children 58

Annex III. Activities for School Children 60

Introduction to the course

Why this course is needed?

School children are the future of our country. Hence it is of vital importance that we inculcate in them good behavior as well as equip them to face the challenges they would meet in the future. We should ensure that they not only achieve physical maturity in good health but are also mentally, psychologically and socially sound and mature. Healthy, responsible, caring and mature adults are the product of good childhood education received in all spheres. As health care professionals and educators it is thus our prime duty to ensure this.

In the present era with heavy academic pressure, most schools fail to provide a good psychosocial environment that is necessitated for good childhood growth. Furthermore the trends present in today's society instill upon our children many negative attributes such as stress, competitiveness, jealousy and anger. All these negative attributes grow within children and the repercussions are seen in their behavior as adults. It thus becomes a vicious cycle. The only place to break this vicious cycle is during the schooling years and the best people to influence our school children is their teacher whom they uphold with utmost respect and admiration.

The psychosocial health promotion of school children – A handbook for teachers training manual has been formulated to train our health and education staff as master trainers. After you successfully complete this course you could go back to your own domains and train the teachers and other educators on the various aspects highlighted through the course so that they could implement activities targeted at improving the psychosocial environment in schools as well as to improve the psychological well being of school children in a targeted and appropriate manner.

Despite the hard work that you would have to carry out in-order to successfully complete this course, we hope that you would find the entire course interesting and rewarding. We hope that through this you would be able to acquire the necessary knowledge skills and attitudes which would enable you to successfully implement programmes to uplift the psychosocial health of school children and adolescents in particular and thereby contribute in producing responsible adults to out Sri Lankan society.

Course Objectives

After the successful completion of this course participants would be able to carry out similar training programmes and train their health staff in the periphery so that they would be able to carry out interventions in order to improve the psychosocial health of school children having a clear understanding of the physiological development experienced by adolescents and school children at large.

The course and the manual

The psychosocial health promotion of school children- A handbook for teachers training manual consists of 18 sessions which could be delivered in any order to suit the target audience. The course coordinator would plan your course to suit your requirements and this order would be given to you in the form of a time table.

This Manual which is the Participant's Manual, includes all the information related to your course. You should keep it with you at all times, as it is the main guide to the course. Each session would first give you an idea on what is expected out of the session through the session objectives. The outline of the session giving the activities that would be carried out for the particular session including the time durations for each of these activities as well as the learning material that is required for the session are also provided. Therefore when preparing to follow as well as to carry out the session you should take special note of these boxes.Each session ends with a box for you to write down the take home messages you received through this session. These would be provided to you as separate sheets of paper. We hope that this would help you to consolidate the knowledge you gained through the session and would serve as a small short note which you could keep in your pocket to be referred at any time. You do not need to take detailed notes during the sessions, though you may find it helpful to make notes of points of particular interest, for example from discussions.Keep your manual with you after the course, and use it when you carry out training programmes in your own areas to train your staff.

Your manual also contains:

- Follow up forms, case studies and take home message sheets for exercises and practical sessions (you will receive separate copies of the forms, case studies and take home message sheets to use for the practical sessions)

Sesssion

01

Training module based on the contents of Chapter 1; Psychosocial Development of Adolescence

This is the first training session of the training module and aims to develop within the trainees an overview as to who an adolescent is. It also aims to create within the participants an overview of the problems, development psychology and psychosocial issues of adolescents.

Objectives

At the end of this session, participants should be able to

- Describe the various charachteristics relevant to the various psychological phases of adolescents
- Understand the problems relevant to the normal development of the adolescent
- Use development psychology to understand the adolescent
- Describe the effects of psychosocial issues on the mental and physical health of adolescents

Session Outline (60 minutes)

Participants are all together for a discussion with the lecturer

I. Introduce the topic (5 minutes)

II. Read pages 1- 4 (20 minutes)

III. Answer the participants questions (5 minutes)

IV. Read pages 4 - 5 (10 minutes)

V. Group discussion (10 minutes)

VI. Presenting discussion points (7 minutes)

VII. Wrapping up (3 minutes)

Material needed

- Psychosocial Health Promotion of school children- A handbook for teachers
- A question box (a small box for questions to be dropped into)
- Small slips of paper to be distributed to the partciapnts to write down their questions
- Flip charts
- Take home message sheet for each participant

I . Introduction (5 minutes)

Make the following points clear

- Adolescents are a vulnerable population
- Propper handeling of adolescents is essential to ensure that they grow up to be healthy adults
- Inorder to ensure this, a proper understanding of their development and problems is essential
- This session would enable use to understand these issues so that we could inturn guide those we train with these issues.

II. Read pages 1-4 (20 minutes)

Request the participants to read out aloud in turn the section on "the psychosocial development and the tasks that characterize the development of adolescents (page 1-4)." After the participant read it, explain to them again the three different phases of adolescents and explain the characteristics in each phase.

III. Answer the participants questions (5 minutes)

Ensure that each row of participants asks at least one question to clarify their doubts. Also pass among the participants a question box and slips of paper. Randomly take out a few questions and answer these as well.

IV. Read pages 4-5(10 minutes)

Request the participants to read out aloud in turn the section on the development psychology, the problems related to normal development, the need for addrsessing these problems, and the effects of psychosocail issues on mental and physical health pages

1. According to Kholberg's theory adolescents should be provided with hypothetical dilemmas where students can explore their feelings and openly discussed their viewpoints critically.

This will increase moral reasoning through group discussions

V. Group discussion (10 minutes)

Divide the participants into groups of five.

The group should discuss on what further point could be presented on the following topics

- Problems of adolescents related to normal development
- Effect of psychosocial issues on Mental and Physical Health of adolescents
- Why addressing these problems are important

You should go around the groups during the group discussions and make sure that all the particiapnts are included in the discussions

VI. Presenting the discussion points (7 minutes)

All members of the group should be prepared to present their discussion findings. Randomly call upon a group member to orally present the discussion findings of the group. The main points to brought up from each group should be written on a flip chart.

VII. Wrap Up (3 minutes)

Randomly select two - three participants and ask them to share their take home messages. After that request all participants to list out the most five important take home messages in the format given below.

Chapter 1; Psychosocial Development of Adolescence

Take home messages

1. ……………………………………………………………………………………………

   ……………………………………………………………………………………………

2. ……………………………………………………………………………………………

   ……………………………………………………………………………………………

3. ……………………………………………………………………………………………

   ……………………………………………………………………………………………

4. ……………………………………………………………………………………………

   ……………………………………………………………………………………………

5. ……………………………………………………………………………………………

   ……………………………………………………………………………………………

Sesssion

02

Training module based on the contents of Chapter 3; Adolescent brain development

This session aims to provide the participants with an overall understanding on how the adolescent brain develops and how the adolescents develop their thinking skills.

Objectives

At the end of this session, participants should be able to

- To understand how the brain attains maturity from birth to adulthood
- To understand the physical, cognitive and social transition experienced by adolescents

Session Outline (90 minutes)

Participants are all together for a discussion with the lecturer

I. Introduce the topic (5 minutes)

II. Lecture presentation (40 minutes)

III. Question and answer session (5 minutes)

IV. Case Scenario Discussions and presentations (35 minutes)

V. Wrapping up the session (5 minutes)

Material needed

- Psychosocial Health Promotion of school children- A handbook for teachers
- A question box (a small box for questions to be dropped into)
- Small slips of paper to be distributed to the partcipants to write down their questions
- Power point presentation
- Case Scenario cards
- Flip charts
- Take home message sheet for each participan

At the beginning of the session all participants sit together for the lecture presentation.

I . Introduce the topic (5 minutes)

State that the maturation of the brain is completetd only at the age of 25 years and therefore the brain of adolescents needs to be trained and pruned inorder to prevent the adolescent from indulging into risky behaviours. You may read pages 13-14 to understand the concept before you speak to your participants.

II. Lecture presentation (40 minutes)

A power point presentation should be made and delivered to the participants. The power point is attached herewith.

III. Question and answer session (5 minutes)

Entertain open questions from the participants pertaining to the lecture. Ensure that at least three questions have been asked.

IV. Case Scenario Discussions and presentations (35 minutes)

The participants should sit in their original groups. The following cases should be divided among the groups. They should discuss the cases within the groups and then present the discussions. Important points that come up in the presentations from each group should be written in the flip charts.

Case 1

"The Perera family has a big decision to make. Mrs. Perera's elderly mother, Mrs. Wimala, has been showing signs of worsening Alzheimer's disease. Her spells of memory loss have become quite disturbing and potentially dangerous. It seems clear that she should no longer live alone in her own house. But where should she go? Of course, one possibility is to have her move in with the Perera family. To make space, the two teenage daughters would have to share a bedroom, which does not really sound appealing to either of them. How can we convince the two daughters the importance of looking after their grand mother?

Case 2

Tharusha is a 14 year old boy, and comes to his mothers office after the school.One of the computers in the office is out of order and he offers his help. Mother asked not to do it as it is an office computer.But he insist that he can do it.How can we help him in decision making?

Case 3

Hiruni is 15 year old school girl and she studied at home alone in the afternoon.One day,despite of her mothers advice she opend the door for an unknown middle aged person who knocked at the door. The man forcefully raped her and no one could hear her call for help.Hiruni got pregnant thereafter.She attempted to commit suicide.

Luckly her mother could prevent her and admitted her to the hospital immediately. Later she delivered the baby and went back to the school and within six months she face the exam and got through.Analyse the situation of Hiruni.

V. Wrapping up the session (5 minutes)

Randomly select two- three participants and ask them to share their take home messages. After that request all participants to list out the most five important take home messages in the format given below.

Chapter 3; Adolescent Brain Development

Take home messages

1. ………………………………………………………………………………………………

   ………………………………………………………………………………………………

2. ………………………………………………………………………………………………

   ………………………………………………………………………………………………

3. ………………………………………………………………………………………………

   ………………………………………………………………………………………………

4. ………………………………………………………………………………………………

   ………………………………………………………………………………………………

5. ………………………………………………………………………………………………

   ………………………………………………………………………………………………

Sesssion

03

Training module based on the contents of Chapter 2; Risk and protective factors for adolescent mental health disorders.

This session aims to provide the participants with an overall understanding on how to promote a healthy mind and positive discipline among adoelescents inoder to create a healthy minds among the adolescents in our community.

Objectives

At the end of this session, participants should be able to

- To identify the risk factors contributing to mental health problems in adolescents
- To identify the positive factors that promote mental health in adolescents
- To understand the multifactor basis of mental disorders in adolescents
- To understand the concept of positive discipline
- To identify the preventive measures of negative behavior in adolescents

Session Outline (90 minutes)

Participants are all together for a discussion with the lecturer

I. Introduce the topic (5 minutes)

II. Read pages 7-9 (10 minutes)

III. Discuss the read material and fill the given venn diagram (15 minutes)

IV. Read pages 9 -10 (10 minutes)

V. Group discussion (10 minutes)

VI. Presenting discussion points (10 minutes)

VII. Read pages 10 -11 (10 minutes)

VIII. Group discussion (5 minutes)

IX. Presenting the discussion points (10 minutes)

X. Wrapping up the session (5 minutes)

Material needed

- Psychosocial Health Promotion of school children- A handbook for teachers
- A question box (a small box for questions to be dropped into)
- 2 Bristol boards with the venn diagram drawn as in the page number 7. Name one board as "Protective Factors" and Other board as " Risk factors"
- 2 x 10 inches sized cards - 30
- Small slips of paper to be distributed to the partcipants to write down their questions
- Flip charts
- Felt pens
- Take home message sheet for each participant

At the beginning of the session divide all the participants into groups of five by asking them to count out from 1-5 aloud. All the number 1s should be grouped together, number 2's should be grouped together etc.

I . Introduce the topic (5 minutes)

State that a range of different physical, psychological, social factors and events to which anyone can be exposed to can cause mental illness.

Also state that the risk factors contributing to mental health problems in adolescents include those at the level of the individual (both biological and psychological), those in the immediate environment (family) and those at the level of the wider environment (community and society at large)

II . Read pages 7-9 (10 minutes)

Request the participants to read out aloud inturn the section on risk factors contributing to mental health problems in adolescents, selected risk and protective factors for mental health of children and adolescents (10 minutes)

III . Discuss the reading material and fill the given venn diagram (15 minutes)

Let the participants read the relevant sections of the teachers guide and write the risk factors for mental health problems in adolescents on provided pieces of cards. Place these cards on the appropriate category of the Venn diagram for risk factors.Follow the same instructions for protective factors for mental health problems in adolescents. The question box should be passed around and a few randomly chosen questions should be answered by the resource person.

IV. **Read pages 9-10 (10 minutes)**

Request the participants to read out aloud inturns the section on positive discipline and effective positive discipline (10 minutes).

V. **Group discussion (10 minutes)**

The already formulated group should discuss on what further point could be presented on the following topics

- With further examples of positive reinforcement, negative reinforcement, positive punishment and negative punishment how the positive discipline parenting and classroom management model could be implemented practically.

(Examples of positive punishments: Ask the child to do a presentation or write a report,

Help in gardening work, Do some shopping,Look after an elderly person at home)

You should go around the groups during the group discussions and make sure that all the participants are included in the discussions

VI. **Presenting discussion points (10 minutes)**

All members of the group should be prepared to present their discussion findings. Randomly call upon a group member to orally present the discussion findings of the group. The main points presented by the groups should be written down on the flip charts.

VII. **Read pages 10 - 11 (10 minutes)**

Request the participants to read out aloud inturn the section on preventive measures of negative behaviour.

VIII. **Group discussion (5 minutes)**

Divide the participants into groups of five.

The group should discuss what measures could be taken to prevent negative behaviours.

You should go around the groups during the group discussions and make sure that all the particiapnts are included in the discussions

IX. **Presenting the discussion points (10 minutes)**

At the beginning of the discussions each group should nominate somebody to present the findings. This person should present the findings at the end of the discussion. The key points presented by the groups should be written down on the flip charts.

X. Wrappping up (5 minutes)

Randomly select two- three participants and ask them to share their take home messages. After that request all participants to list out the most five important take home messages in the format given below.

Chapter 2; Risk and protective factors for adolescent psychosocial Development

Take home messages

1. ……………………………………………………………………………………………...........…
   ……………………………………………………………………………………………...…
2. ……………………………………………………………………………………………...……
   ……………………………………………………………………………………………..………
3. ……………………………………………………………………………………..……………
   ………………………………………………………………………………………..……………
4. …………………………………………………………………………………..………………
   ………………………………………………………………………………………………………
5. ………………………………………………………………………………………………………
   ………………………………………………………………………………………………………

Sesssion

04

Training module based on the contents of Chapter 4;Stress and Educational/Academic Problems in Adolescence

This session aims to provide the participants with an overall understanding on the stress experienced by school children, its causes and its after effects.

Objectives

At the end of this session, participants should be able to

- To understand stress and its various types
- To understand why stresss occours in children
- To identify a stressed child
- Understand the complications of stress
- Understand how to cope with and help a child who is stressed
- Understand the common educational/academic problems in adolescence
- Understand how studies and extra curricular activities should be balanced
- Effects of teacher student relationships

Session Outline (120 minutes)

I. Introduce the topic (5 minutes)

II. Lecture presentation (45 minutes)

III. Question and answer session (5 minutes)

IV. Role Play (40 minutes)

V. Group Discussion (20 minutes)

VI. Wrapping up the session (05 minutes)

Material needed

- Psychosocial Health Promotion of school children- A handbook for teachers
- A question box (a small box for questions to be dropped into)
- Small slips of paper to be distributed to the partcipants to write down their questions
- Power point presentation
- Role Play cards
- Take home message sheet for each participant

At the beginning of the session all participant would be seated together in order to listen to a lecture.

I. Introduce the topic (5 minutes)

Indicate to the participants that stress is the reaction mounted by our brain and body when confronted with an unusually demanding situation. State that in this session they would discuss some brief common behavioural problems related to academic performances and would also be able to obtain an experience on these issued through role plays.

II. Lecture presentation (45 minutes)

A power point lecture should be delivered to the participants. The power point presentation is attached herewith.

III. Question and answer session (5 minutes)

The question box should be passed along during the lecture. After the lecture any open questions as well as a few selected questions from the box should be answered.

IV. Role Play (40 minutes)

The participants should devide into three groups. Each group would be given a role play with ten minutes preparation time. Thereafter each group would be given a chance to present their role play. Each time the non performing group should critically comment on the performance.

Role Play 1

11 years old Suvini entered to a National School in the city, after scoring high marks for the scolarship exam, from a rural School. She was boarded in the school hostel since the school is far away from the home. Suvini couldn't bear the loneliness created due to seperation from her parents. Everyday during the interval, she calls her mother from the telephone booth in the school, cries and requests to take her home.

Role Play 2

19 year old Supun was awaiting to sit for the GCE/AL exam in Science Stream. He was the best student in the school and parents, teachers, relatives and friends kept high hopes about his performances. Friends called him "Dr. Supun" Supun couldn't

tolerate this and suddenly became violent and complaint that he cannot remember any thing. He also refused to sit for GCE/AL., this year.

Role Play 3

Saveen who is a 16 year old boy complaint of lack of sleep , difficulty in memorizing . He hates the vast curricular and he takes tution because of difficulty in understanding. He consulted the school counsellor and discuss the issue.

V. Group Discussion (20 minutes)

Divide the participants into four groups.

The group should discuss ways of anxiety reduction techniques in schools study techniques and how to face exams.

You should go around the groups during the group discussions and make sure that all the participants are included in the discussions.

At the end of the discussion each group should present.

VI. Wrapping up the session (5 minutes)

Randomly select two- three participants and ask them to share their take home messages. After that request all participants to list out the most five important take home messages in the format given below.

Chapter 4;Stress and Educational/Academic Problems in Adolescence

Take home messages

1. ……………………………………………………………………………………………
……………………………………………………………………………………………
2. ……………………………………………………………………………………………
……………………………………………………………………………………………
3. ……………………………………………………………………………………………
……………………………………………………………………………………………
4. ……………………………………………………………………………………………
……………………………………………………………………………………………
5. ……………………………………………………………………………………………
……………………………………………………………………………………………

Sesssion

05

Training module based on the contents of Chapter 5; Mental Health Problems of adolescents and chidlren

This session aims to provide the participants with an overall understanding on the common mental health issues of children.

Objectives

At the end of this session, participants should be able to

- To identify the different types of mental health issues
- To understand the charachteristics of each of these issues
- To understand the role of the teachers in handeling children with each of these mental health issues

Session Outline (60 minutes)

Participants are all together for a discussion with the lecturer

I. Introduce the topic (3 minutes)

II. Lecture presentation (50 minutes)

III. Question and answer session (5 minutes)

V. Wrapping up the session (2 minutes)

Material needed

- Psychosocial Health Promotion of school children- A handbook for teachers
- A question box (a small box for questions to be dropped into)
- Small slips of paper to be distributed to the partcipants to write down their questions
- Power point presentation
- Take home message sheet for each participant

At the beginning of the session all participants would be seated together in order to listen to a lecture.

I. Introduce the topic (3 minutes)

State that the main mental health issues do not exist separately but are interconnected with each other. The mental well being of the child should be in a favourable state inorder for the child to leave as an educated young adult.

II. Lecture presentation (50 minutes)

A brief overview of the mental health issues should be presented in a power point presentation. The power point presentation is attached herewith.

III. Question and answer session (5 minutes)

The question box should be passed around the participants during the lecture and the reading session. The resource person should answer randomly chosen questions as well as should answer any open questions asked by the participants.

V. Wrapping up the session (2 minutes)

Randomly select two- three participants and ask them to share their take home messages. After that request all participants to list out the most five important take home messages in the format given below.

Chapter 5; Mental Health Problems of adolescents and children

Take home messages

1. ……………………………………………………………………………………………

……………………………………………………………………………………………

2. ……………………………………………………………………………………………

……………………………………………………………………………………………

3. ……………………………………………………………………………………………

……………………………………………………………………………………………

4. ……………………………………………………………………………………………

……………………………………………………………………………………………

5. ……………………………………………………………………………………………

……………………………………………………………………………………………

Sesssion

06

Training module based on the contents of Chapter 6;Mental Health Aspects of Sexual and Reproductive Health Problems in Adolescence

This session aims to provide the participants with an overall understanding on the importance of educating adolescents on sexual and reproductive health

Objectives

At the end of this session, participants should be able to

- Describe the importance of sexual and reproductive health and its association with the mental health of adolescents
- Define Puberty
- Understand the psychosocial changes that occour during puberty
- Describe the female and male reproductive health system
- Understand special features specific to females and males
- Understand the process of fertilization
- Understand what is meant by sexual orientation
- Describe sexual bullying and harassment
- Understand what is meant by sexually transmited infections

Session Outline (90 minutes)

Participants are all together for a discussion with the lecturer

I. Introduce the topic (5 minutes)

II. Deliver the lecture (45 minutes)

III. Open discussion with the participants (35 minutes)

IV. Wrapping up the session (5 minutes)

Material needed

- Psychosocial Health Promotion of school children- A handbook for teachers
- A question box (a small box for questions to be dropped into)
- White Board and Marker Pens
- Small slips of paper to be distributed to the partcipants to write down their questions
- Power point presentation
- Take home message sheet for each participant

At the beginning of the session all participants are seated together .

I. Introduce the topic to the participants (5 minutes)

State that the body image is a person's opinions, thoughts, and feelings about his or her own body and physical appearance. Having a positive body image means feeling pretty satisfied with the way they look, appreciating the body for its capabilities and accepting its imperfections.

However, state that although body image is just one part of the self-image, during the teen years, and especially during puberty, it can be easy for a person's whole self image to be based on how his/her body looks.

Say that this is because adolescents'bodies are changing so much during this time that they can become the main focus of their attention. State that a change in adolescents' body can be tough to deal with emotionally. Some don't feel comfortable in their changing bodies and can feel as if they don't know who they are anymore. Some go into puberty not feeling too satisfied with their body or appearance to begin with. (Example, over-weight, obesity, short body or disabled).Therefore puberty may add to their insecurities.

II. Deliver the lecture (45 minutes)

The power point slides are attached herewith. Before the lecture commences tell the participants that a question box would be passed around during the lecture. Hand the participants slips to jot down their questions. Ask the participants to drop in their slips into the box.

While the lecture is being carried out the question box should be passed to the participants.

III. Open discussion with the participants (35 minutes)

Each group should have a white board and a marker pen. The facilitator would trigger the discussion by answering few questions from the box. The facilitator would also pose questions to the participants with the aim of stimulating an open discussion.

IV. Wrapping up the session(5 minutes)

Randomly select two participants and ask them to share their take home message. Thereafter ask the participants to write down their take home message on the sheet provided.

Chapter 6;Mental Health Aspects of Sexual and Reproductive Health Problems in Adolescence

Take home messages

1. ……………………………………………………………………………………………...........…

   ……………………………………………………………………………………………..…

2. ……………………………………………………………………………………………..……

   ……………………………………………………………………………………………..………

3. ……………………………………………………………………………………………..………

   ……………………………………………………………………………………………..………

4. ……………………………………………………………………………………………..………

   ……………………………………………………………………………………………………

5. ………………………………………………………………………………………………………

   ……………………………………………………………………………………………………

Sesssion

07

Training module based on the contents of Chapter 7 on Impacts of Bullying at School

This session aims to provide the participants with an overall understanding on the importance of preventing bullying among school children.

Objectives

At the end of this session, participants should be able to

- Understand the meaning of bullying
- Learn to differentiate the difference between bullying and fighting
- Develop mechanisms to prevent bullying in the community
- Understand the short term and long term effects of bullying

Session Outline (130 minutes)

Participants are all together for a discussion with the lecturer

I. Buzz group discussion on what is meant by Bullying (10 minutes)
II. Read the pages 55-57 from the psychosocial guide (10 minutes)
III. Lecture Presentation (10 minutes)
IV. Group project on how to prevent bullying among school children (45 minutes)
V. Presentation of group projects (40 minutes)
VI. Read pages 57-59   from the psychosocial guide (10 minutes)
VII. Wrapping up the session (5 minutes)

Material needed

- Psychosocial Health Promotion of school children- A handbook for teachers
- Power point Presentation on "bulling"
- Four Bristle Boards
- Four sets of coloured marker pens
- Take home message sheet for each participant
- Follow up forms

At the beginning of the session all participants are seated together

I. Buzz group discussion on what is meant by Bullying (10 minutes)

A buzz group should be initiated to find out what the participants mean by the word "Bullying" Ask the participants to pair up in either twos or threes with the person/ persons seated next to them and disucuss what they mean by the word bullying. Please inform the participants that they would be called upon to randomly present what they discuss in their buzz groups. After 7 minutes of buzz groups randomly select participants and ask them to tell the group what was dicussed.

This activity would trigger what the word Bullying means among the participants.

II. Read the pages 55-57 from the psychosocial guide (10 minutes)

III. Lecture Presentation (10 minutes)

An Overview of Bulling Should be presented in a Powerpoint Presentation, given to you.

IV. Group project on how to prevent bullying among school children (45 minutes)

Divide the group into four by asking people from same MOH areas/RDHS areas (depending on who is present) to group together. Then hand over a bristol board and a set of pens to each group and ask them to design a programme to prevent bullying to be implemented in schools in their area.They should also be ready to present their project.

V. Presentation of group projects (40 minutes)

Each group should be given 10 minutes to present their project. Discussion should take place for about 5 minutes after each presentation.

VI. Read pages 57-59 from the psychosocial guide (10 minutes)

VII. Wrapping up the session (5 minutes)

After the reading from the book has been completed ask the groups to discuss any modifications they wish to carry out in their project. Then appoint a person from each MOH area/RDHS area to monitor the programmes that are implemented to prevent bullying in schools in his/her MOH area/RDHS area during the next six months. Handover the follow up form (shown below) to each such person in a MOH area/ RDHS area.Indicate that this should be filled and returned to the school health unit (FHB). State that only when all such forms are received timely in six months would the training of trainers certificate be issued to the participants.

Thereafter ask randomly selected participants on the key issues that were discussed during the session. Thereafter the partcipants could fill in their take home message form.

Chapter 7 :Impacts of Bullying at School

Take home messages

1. ………………………………………………………………………………………………

………………………………………………………………………………………………

2. ………………………………………………………………………………………………

………………………………………………………………………………………………

3. ………………………………………………………………………………………………

………………………………………………………………………………………………

4. ………………………………………………………………………………………………

………………………………………………………………………………………………

5. ………………………………………………………………………………………………

………………………………………………………………………………………………

Follow up form for prevention of Bullying and Violence at School

Name of the MOH area :

Name of the RDHS area:

Date :

Name of School which activity was conducted	Description of activity	Name of staff involved in implementing the programme	Comments of School Principal

Name of MOH: ………………………………………………………….

Signature of MOH: ……………………………………… Date :…………………………

OR

Name of RDHS: ………………………………………………….

Signature of RDHS: ………………………………………… Date: ………………………..

Sesssion

08

Training module based on the contents of Chapter 8 ;Violence and its consequences in adolescence

This session aims to provide the participants with an overall understanding on the importance of preventing violence among school children.

Objectives

At the end of this session, participants should be able to

- Define violence in schools
- Understand the burden of violence in schools
- Understand the factors associated with school violence
- Describe the after effects of school violence
- Design a programme to prevent violence in schools in their area

Session Outline (145 minutes)

Participants are all together for a discussion with the lecturer

I . Buzz group discussion on what is meant by violence in schools (10 minutes)
II. Introduction by the facilitator (10 minutes)
III. Read the pages 61-64 from the psychosocial guide (20 minutes)
IV. Group project on how to prevent violence among school children (45 minutes)
V. Presentation of group projects (40 minutes)
VI. Read pages 64-67 from the psychosocial guide (15 minutes)
VII. Wrapping up the session (5 minutes)

Material needed

- Psychosocial Health Promotion of school children- A handbook for teachers
- Four bristol boards
- Four sets of coloured marker pens
- Take home message sheet for each participant
- Follow up forms

At the beginning of the session all participants are seated together .

I. Buzz group discussion on what is meant by violence in schools (10 minutes)

As before a buzz group should be initiated to find out what the participants mean by "Violence in Schools." Ask the participants to pair up in either twos or threes with the person/persons seated next to them and discuss what they mean by "Violence in Schools." Please inform the participants that they would be called upon to randomly present what they discuss in their buzz groups. After 7 minutes of buzz groups randomly select participants and ask them to tell the group what was dicussed.

This activity would trigger thoughts of violence in schools among the participants.

II. Introduction by the facilitator (10 minutes)

Following the buzz group discussion introduce what is meant by Violence in Schools to the participants.State that School violence is a violent event that occurs in school, on the way to school or at a school sponsored event, or during school sponsored event. Say that a student can be a victim, a perpetrator or witness.

Mention also that various types of violence can occur in schools; physical, verbal and non-verbal or gestural forms and sexual. Stress to the participants that it can badly affect the physical, mental and social wellbeing of students. State also that some violent acts such as bullying, slapping or hitting could cause more emotional harm than physical harm while other forms, such as gang violence and assault (with or without weapons), can lead to serious injury or even death.

III. Read the pages 61-64 from the psychosocial guide (20 minutes)

IV. Group project on how to prevent violence among school children (45 minutes)

Divide the group into four by asking people from same MOH areas /RDHS areas (depending on who is present) to group together. Then hand over a Bristol board and a set of pens to each group and ask them to design a programme that could be implemented in schools in their area in oder to prevent violence in these schools.They should also be ready to present their project.

V. Presentation of group projects (40 minutes)

Each group should be given 10 minutes to present their project. Discussion should take place for about 5 minutes after each presentation.

VI. Read pages 64-67 from the psychosocial guide (15 minutes)

VII. Wrapping up the session (5 minutes)

After the reading from the book has been completed ask the groups to discuss any modifications they wish to carry out in their project. Then appoint a person from each MOH area/RDHS area to monitor the programmes that are implemented to prevent violence in schools in his/her MOH area/RDHS area during the next six months. Handover the follow up form (shown below) to each such person in a MOH area/ RDHS area. Indicate that this should be filled and returned to the school health unit. State that only when all such forms are received timely in six months would the training of trainers certificate be issued to the participants.

Thereafter ask randomly selected participants on the key issues that were discussed during the session. Thereafter the partcipants could fill in their take home message form.

Chapter 8: Violence and its consequences in adolescence

Take Home Messages

1. ……………………………………………………………………………………………

……………………………………………………………………………………………

2. ……………………………………………………………………………………………

……………………………………………………………………………………………

3. ……………………………………………………………………………………………

……………………………………………………………………………………………

4. ……………………………………………………………………………………………

……………………………………………………………………………………………

5. ……………………………………………………………………………………………

……………………………………………………………………………………………

Follow up form for prevention of Bullying and Violence at School

Name of the MOH area :

Name of the RDHS area:

Date :

Name of School which activity was conducted	Description of activity	Name of staff involved in implementing the programme	Comments of School Principal

Name of MOH: …………………………………………………………

Signature of MOH: ……………………………………… Date :…………………………

OR

Name of RDHS: …………………………………………………

Signature of RDHS: ………………………………………… Date: ………………………..

Sesssion

09

Training module based on the contents of Chapter 9;Effects of Child Abuse on childhood and adolescence

This session aims to provide the participants with an overall understanding on the importance of preventing child abuse among school children.

Objectives

At the end of this session, participants should be able to

- Understand what is meant by child abuse
- Describe the basic types of child abuse
- Understand what makes people abuse children
- Understand why children don't speak out about child abuse
- Undertsand why society has a closed eye to child abuse
- Describe the myths associated with child abuse
- Describe the factors associated with child abuse
- Describe what to do when they come in contact with a victim of child abuse

Session Outline (100 minutes)

Participants are all together for a discussion with the lecturer

I. Activity to identify what is and what is not child abuse (10 minutes)

II. Read the pages 69-71 from the psychosocial guide (15 minutes)

III. Read the pages 72-74 from the psychosocial guide (10 minutes)

IV. Case Scenario Discussion (45 minutes)

V. Activity to present the methods of preventing child abuse (15 minutes)

VI. Wrapping up the session (5 minutes)

Material needed

- Psychosocial Health Promotion of school children- A handbook for teachers
- Two White Boards
- Case Scenarios
- Coloured marker pens
- Four Bristol Boards
- 20 blank half sheets
- 20 Magnets
- Take home message sheet for each participant

I. Activity to identify what is and what is not child abuse (10 minutes)

All the participants would be seated together in one hall. Ask each participant to say 1,2etc in order. Thereafter divide the participants into two groups based on whether they said number 1 or number 2.

Ask them to form two circles. Name the two groups as 1 and 2. Ask group number 1 to discuss within the group and formulate a list of things which they feel should be included as child abuse. Ask group 2 to discuss within the group and formulate a list of things which they feel should not be categorized as child abuse. After 20 minutes ask one person from each group to come up and write down separately on each of the two white boards the formulated lists. Therefore one white board would have what they think of as child abuse and the other white boad would have what they feel is not child abuse.

II. After this the participants should read out a loud pages 69-71 from the Psychosocial hand book(15 minutes)

After reading, one representative from each group to come up and modify the lists made by the participants with the knowledge gained from the book. These modifications should be carried out with a different coloured marker pen. Group members should actively participate in modifying this list and the facilitator should facilitate the participation of all group members.

III. Read the pages 72-74 from the psychosocial guide (10 minutes)

IV. Case Scenario Discussion (45 minutes)

Divide the group into four. Hand over a case study to each of the four groups and ask them to discuss how they would have handled each case if the victim had been brought to them.

Case Study 1

Lalani was 14 when she met her first 'boyfriend'. He – in his 30s – bought her presents, picked her up in his car, told her he loved her. But he soon changed. He became

violent and before long was forcing her to have sex with his friends. Then, like a toy, Lalani was passed on, shipped around the country and raped by countless men. "I got taken to flats. I don't know where they were and men would be brought to me," she said. "I was never given any names and I don't remember their faces."

Case Study 2

Sonali was 13 when she met a boy who said he was 18, at her cousin's 21st birthday party. "I thought he was gorgeous, it was really exciting," she said.

At first he treated her well, but soon he started to control her, isolating her from her family. Asked if he hit her often she said: "Just when I wouldn't do something he wanted me to do" She paused, then added in a small voice: "So, yeah, often.The man didn’t do any sexual harressment but hit her always for not giving consent. He didn’t want her to do anything without his knowledge.He checked her phone regularly. He also scold her infront of others.Sonali told her problem to the school councelling teacher and the teacher reffered her to you.

Case Study 3

Sayuri was a 17 year old girl from a rural area and studied in a leading school in the city. She was boarded in one of her relatives house. Once she was alone with a 55 year old uncle in that house. Unfortunately Sayuri was raped by her uncle on that day.

Case Study 4

11 year old Sahan started to travel in the school bus alone very recently. A boy in the A/Level class always ask him to sit beside him and shows prone vedios from his phone when ever he meet him in the school bus.This has become a nuisance to Sahan but he can’t tell any body since the A/L student threatened him.

Facilitator should tell the participants the goals of any effective response to suspected child abuse at the end of the discussion

- Reffer page 74 from the psycosocial guide:

V. Activity to present the methods of preventing child abuse (15 minutes)

Give 20 A4 half sheets randomly to the participants. Ask them to write a method of preventing child abuse on their sheet and put them on the white board using magnets.

VI. Wrapping up the session (5 minutes)

Ask randomly selected participants on the key issues that were discussed during the session. Thereafter the partcipants could fill in their take home message form.

Chapter 9 :Effects of Child Abuse on childhood and adolescence

Take Home Messages

1. ………………………………………………………………………………………………………..........…
   ………………………………………………………………………………………………………...…
2. ………………………………………………………………………………………………...……
   ………………………………………………………………………………………………..………
3. ………………………………………………………………………………………….…………
   ………………………………………………………………………………………..……………
4. ………………………………………………………………………………………..……………
   ……………………………………………………………………………………………………
5. ……………………………………………………………………………………………………
   ……………………………………………………………………………………………………

Sesssion

10

Training module based on the contents of Chapter 10;Chronic physical illnesses – the effects on mental health

This session aims to provide the participants with an overall understanding on the impact of the mental health of school children due to chronic disease.

Objectives

At the end of this session, participants should be able to

- Understand that children with chronic illnesses may face various kinds of stresses
- Describe the problems faced by such children at school
- Describe how health/education professionals could help such children
- Describe why such children may be non compliant

Session Outline (50 minutes)

Participants are all together for a discussion with the lecturer

I. Read the pages 77 - 80 from the psychosocial guide (20 minutes)

II. Group Discussion (15 minutes)

III. Read pages 80-81 from the psychosocial guide (10 minutes)

IV. Wrapping up the session (5 minutes)

Material needed

- Psychosocial Health Promotion of school children- A handbook for teachers
- Take home message sheet for each participant

I. **Read the pages 77 - 80 from the psychosocial guide (20 minutes)**

II. **Group Discussion (15 minutes)**

Ask the participants to group up with the people next to them and discuss as to why some teenagers/adoelscents do not comply with medical treatment

III. **Read pages 80-81 from the psychosocial guide (10 minutes)**

IV. **Wrapping up the session (5 minutes)**

Ask three participants to volunteer and say one key issue each that was discussed during the session. Thereafter ask the partcipants to fill in their take home message form.

Chapter 10; Chronic physical illnesses – the effects on mental health

Take Home Messages

1. ……………………………………………………………………………………………

……………………………………………………………………………………………

2. ……………………………………………………………………………………………

……………………………………………………………………………………………

3. ……………………………………………………………………………………………

……………………………………………………………………………………………

4. ……………………………………………………………………………………………

……………………………………………………………………………………………

5. ……………………………………………………………………………………………

……………………………………………………………………………………………

Sesssion

11

Training module based on the contents of Chapter 11;Impacts of Information Technology and Media on adolescent psychosocial wellbeing

This session aims to provide the participants with an overall understanding on the importance of educating adolescents and their parents on how to engage in safe media practices

Objectives

At the end of this session, participants should be able to

- Obtain an overall understanding on media and social media
- Describe the positive and negative effects of media
- Describe what the teacher could do to promote safe media practices among school children

Session Outline (155 minutes)

Participants are all together for a discussion with the lecturer

I. Lecture discussion (120 minutes)

II. Read pages 86 to 87 from psycho social guide and prepare posters (30 minutes)

III. Wrapping up the session (5 minutes)

Material needed

- Power point Presentation
- Take home message sheet for each participant
- 3 bristol boards
- Coloured felt pens

At the beginning of the session all participants are seated together.

I. Lecture discussion (120 minutes)

An overview of impacts of Information technology and media on adolescent psycho social wellbeing should be presented in a power point presentation. The power point presentation is attached herewith.

II. Read pages 86 to 87 from psycho social guide (30 minutes)

Devide the group into 3 devide the following topics among them and ask to prepare one poster on each topic.

1. Teachers role in safe use of media
2. Parents role in safe use of media
3. Health care workers role in safe use of media

III. Wrapping up the session (5 minutes)

Randomly select two participants and ask them to share their take home message. Thereafter ask the participants to write down their take home message on the sheet provided.

Chapter 11; Impacts of Information Technology and Media on adolescence

Take home messages

1. ……………………………………………………………………………………………
……………………………………………………………………………………………
2. ……………………………………………………………………………………………
……………………………………………………………………………………………
3. ……………………………………………………………………………………………
……………………………………………………………………………………………
4. ……………………………………………………………………………………………
……………………………………………………………………………………………
5. ……………………………………………………………………………………………
……………………………………………………………………………………………

Sesssion

12

Training module based on the contents of Chapter 12;Health Issues on Vulnerable Groups

This session aims to provide the participants with an overall understanding on how to address the health issues of vulnerable adolescents

Objectives

At the end of this session, participants should be able to

- Identify vulnerable students within their communities
- Identify the common health issues present among vulnerable adolescents
- Identify their role in assisting vulnerable school children and adolescents

Session Outline (70 minutes)

Participants are all together for a discussion with the lecturer

I. Read pages 91-92 (5 minutes)

II. Developing a plan of activities to prevent health issues in vulnerable groups (20 minutes)

III. Presenting the plan of activities (40 minutes)

IV. Wrapping up the session (5 minutes)

Material needed

- Psychosocial Health Promotion of school children- A handbook for teachers
- Flip charts
- Marker Pens
- Take home message sheet for each participant

At the beginning of the session all participants are seated together

I. Read pages 91-92 (5 minutes)

The facilitator should pinpoint and ask a participant to read. The participant should be stopped after small sections of reading and another chosen so that all would be involved. During such breaks the facilitator should describe the contents in simple words

II. Developing a plan of activities to prevent health issues in vulnerable groups (20 minutes)

Divided the participants into 8 groups. Ask the participants to prepare a plan of activities to prevent health issues in the below mentioned vulnerable groups.

i. Children and young people living in relative poverty

ii. Children in care

iii. Children and young people at risk because of mental health problems

iv. Children and young people with learning difficulties and / or disabilities

v. Young carers

vi. Children and young people who are victims of neglect and abuse

vii. Young offenders

viii. Children and young people living in temporary accommodations including IDP camps and conflict affected areas

Write the points on a flip chart and appoint a group member to present.

III. Presenting the plan of activities (40 minutes)

A group member from each group should present their plan of activities. All groups should present. A few minutes of discussion should follow each presentation

IV. Wrapping up the session (5 minutes)

Randomly select two participants and ask them to share their take home message. Thereafter ask the participants to write down their take home message on the sheet provided.

Chapter 12 ;Health Issues on Vulnerable Groups

Take Home Messages

1. ………………………………………………………………………………………………..........…

………………………………………………………………………………………………..…

2. ……………………………………………………………………………………………...……

…………………………………………………………………………………………..………

3. ………………………………………………………………………………………..…………

……………………………………………………………………………………..……………

4. …………………………………………………………………………………...………………

……………………………………………………………………………………………………

5. ………………………………………………………………………………..…………………

……………………………………………………………………………………………………

Sesssion

13

Training module based on the contents of Chapter 13; School Children with Substance Abuse, a Practical Approach for Teachers

This session aims to provide the participants with an overall understanding on how to address the health issues related to substance abuse among school children.

Objectives

At the end of this session, participants should be able to

- Name the substances commonly abused by school children
- Understand the impact of substance abuse among school children
- Understand the effects of addiction
- Understand why children abuse substances
- Understand how drugs could be inadvertently be promoted
- Understand how to prevent substance abuse among school children

Session Outline (125 minutes)

Participants are all together for a discussion with the lecturer

I. Read pages 99-104 (20 minutes)

II. Discussion among facilitator and participants on the newer methods of prevention of drug abuse (30 minutes)

III. Role play (30 minutes)

IV. Discuss the activities for prevention of substance abuse among school children (20 minutes)

V. Presenting the activities (20 minutes)

VI. Wrapping up the session (05 minutes)

Material needed

- Psychosocial Health Promotion of school children- A handbook for teachers
- Flip charts
- Marker Pens
- Power point presentation
- Take home message sheet for each participant

At the beginning of the session all participants are seated together

I. Read pages 99-104 of the psycho social health promotion guide. (20 minutes)

II. Discussion among facilitator and participants on the newer methods of prevention of drug abuse (30 minutes)

The facilitator should discuss the newer methods of prevention of drug abuse. The power point presentation needed is attached herewith.

III. Role play (30 minutes)

Divide the participants into two groups and ask them to select a student and a teacher from each group. Allocate one of the following role plays to each group ask them to do the role play.

i. Counselling a student who is addicted to smoking cigarettes

ii. Counselling a student who is addicted to alcohol

Ask the other participants to express their ideas critically about the presentations.

IV. Discuss the activities for prevention of substance abuse among school children (20 minutes)

Divide the group into three and ask them to discuss the activities for the prevention of substance abuse among school children. Write the points on a flip chart and appoint a group member to present.

concider the methods to reduce availability of substances as well.

V. Presenting the activities (20 minutes)

Each group should present their activities. After presentation the rest of the class should discuss the pros and cons of the presentation.

VI. Wrapping up the session (5 minutes)

Randomly select two participants and ask them to share their take home messages. Thereafter ask the participants to write down their take home message on the sheet provided.

Chapter 13; School Children with Substance Abuse, a Practical Approach for Teachers

Take Home Messages

1. ……………………………………………………………………………………………

……………………………………………………………………………………………

2. ……………………………………………………………………………………………

……………………………………………………………………………………………

3. ……………………………………………………………………………………………

……………………………………………………………………………………………

4. ……………………………………………………………………………………………

……………………………………………………………………………………………

5. ……………………………………………………………………………………………

……………………………………………………………………………………………

Sesssion

14

Training module based on the contents of Chapter 14 on Teacher's Role in Handling Difficult Children

This session aims to provide the participants with an overall understanding on how to handle difficult children at school

Objectives

At the end of this session, participants should be able to

- Understand the difficult behaviours that would present at school
- Understand how to tackle children with difficult behaviours at school

Session Outline (35 minutes)

Participants are all together for a discussion with the lecturer

I. Read pages 113-115 (10 minutes)

II. Small Group Discussion (20 minutes)

III. Wrapping up the session (5 minutes)

Material needed

- Psychosocial Health Promotion of school children- A handbook for teachers
- Take home message sheet for each participant
- Four Facilitators – One for each group

The participants could be randomly divided into four groups for this activity.

I. Read pages 113-115 (10 minutes)

Within the groups they should read out aloud pages 113-115.

II. Small Group Discussion (20 minutes)

Within the same groups the participant should share their experiences of the difficult children they have had to deal with in the past. The facilitators should go round the groups to ensure that all participate and guide the discussion within the groups.

Discuss how to control the behaviour of a hyperactive child and a temper tantrum of a autistic child.

III. Wrapping up the session (5 minutes)

Thereafter ask randomly selected participants on the key issues that were discussed during the session. Thereafter the participants could fill in their take home message form.

Chapter 14;Teacher's Role in Handling Difficult Children

Take Home Messages

1. ……………………………………………………………………………………………...........…

……………………………………………………………………………………………………...…

2. ……………………………………………………………………………………………...………

……………………………………………………………………………………………...…………

3. ……………………………………………………………………………………………..…………

……………………………………………………………………………………………...……………

4. ……………………………………………………………………………………………..…………

……

5. ……

……

Sesssion

15

Training module based on the contents of Chapter 15; Mental Health Promotion at School

This session aims to provide the participants with an overall understanding on the mental health promotion of school children

Objectives

At the end of this session, participants should be able to

- Have a good understanding on the National School Health Programme and its role in promoting mental health among school children
- Identify the factors affecting mental health within the school context
- Identify a student with mental health problems
- Identify how teachers could promote the well being of the school community

Session Outline (55 minutes)

Participants are all together for a discussion with the lecturer

I. Read pages 117-118 (5 minutes)

II. Open Group Discussion (20 minutes)

III. Read pages 118-120 (5 minutes)

IV. Think Pair Share activity (20 minutes)

V. Wrap up the session (5 minutes)

Material needed

- Psychosocial Health Promotion of school children- A handbook for teachers
- White Board
- Marker Pens
- Take home message sheet for each participant

At the beginning of the session all participants are seated together

I. Read pages 117-118 (5 minutes)

The facilitator should randomly ask a member of the group to read out pages 117-118. If the facilitator wishes he could ask two persons to read taking turns.

II. Open Group Discussion (20 minutes)

The facilitator should divide the white board into two parts. One should be for factors which promote the mental health of school children. The other should be for the risk factors. The participants should be asked to contribute to fill these two sides and explain.

III. Read pages 118-120 (5 minutes)

Thereafter randomly chosen participants should be asked to read from pages 118-120. Following that any points that were left out during the open discussion should be added on the white board.

IV. Think Pair Share activity (20 minutes)

Divide the participants into four groups. Ask them to take 3 minutes and to think by themselves how they could promote mental well being among the school community. Thereafter ask them to pair up with the person next to them and share their views. Please inform the participants that they would be called upon randomly to present what they discussed in their groups. After 7 minutes of discussion and ask the randomly selected participants to present what was discussed.

Following this activity ask the participants to read page 121 within the group.

V. Wrap up the session (5 minutes)

Ask randomly selected participants on the key issues that were discussed during the session. Thereafter the participants could fill in their take home message form.

Chapter 15; Mental Health Promotion at School

Take Home Messages

1. ………

………

2. ………

………

3. ………

………

4. ………

………

5. ………

………

Sesssion

16

Training module based on the contents of Chapter 16; Life Skills – A way out for mental health problems

This session aims to provide the participants with an overall understanding on the importance of life skills to overcome challenges in day- to- day life.

Objectives

At the end of this session, participants should be able to

- Define what is meant by Life Skills
- Name the 10 Life Skills
- Understand the importance of life skills in the life of a school child
- Describe each life skill and understand how to demonstrate these life skills to school children
- Identify the ways of monitoring life skills.

Session Outline (5 hours and 45 minutes)

Participants are all together for a discussion with the lecturer

i. Activity to name the life skills (5 minutes)

ii. Debate on the importance of life skills for adolescents. (30 minutes)

iii. Read page 125 from the psycho social guide (5 minutes)

iv. Case scenario discussions and presentations (5 hours)

v. Wrapping up the session (5 minutes)

Material needed

- Psychosocial Health Promotion of school children- A handbook for teachers
- Paper ball
- Flip chart
- Marker Pens
- Take home message sheet for each participant

I. Activity to name the life skills (5 minutes)

Pass a paper ball to a participant. Ask her /him to name a life skill. Again pass the paper ball to another participant and continue naming the life skills like this.

II. Debate on the importance of life skills for adolescents. (30 minutes)

Divide the participants into two groups ask each group to select 3 members from each group as a debating team. The 2 teams should debate on whether life skills are important for adolescents or not. Give the topic to each group after tossing a coin.

III. Read page 125 from the psycho social guide (5 minutes)

Ask the participants to read page 125 from psychosocial guide on the problems faced by children/youths when they have poor life skills.

IV. Case scenario discussions and presentations (5 hours)

- Divide the participants into 7 groups.Give 2 case scenarios per group. (case scenarios are given in the Psychosocial Health Promotion of school children - A handbook for teachers) Let them discuss the 2 case scenarios for 20 minutes within the group. Important points came up in the discussion should be written on flip charts. Each group should present their discussions. Utilize 10 minutes for each presentation. Facilitator should comment after each case scenario for 10 minutes.

V. Wrap up the session (5 minutes)

Ask randomly selected participants on the key issues that were discussed during the session. Thereafter the participants could fill in their take home message form.

Chapter 16; Life Skills – A way out for mental health problems Take Home Messages

1. ……………………………………………………………………………………………………
……………………………………………………………………………………………………
2. ……………………………………………………………………………………………………
……………………………………………………………………………………………………
3. ……………………………………………………………………………………………………
……………………………………………………………………………………………………
4. ……………………………………………………………………………………………………
……………………………………………………………………………………………………
5. ……………………………………………………………………………………………………
……………………………………………………………………………………………………

Sesssion

17

Training module based on the contents of chapter 17:on Mindful School (Sati Pasala) A Newly Recognized Intervention to Improve Psychosocial Wellbeing

This session aims to provide the participants with an overall understanding on the concept of a mindful school and its importance for the psycho social wellbeing of adolescents.

Objectives

At the end of this session, participants should be able to

- Understand what is meant by mindfulness and mindful school.
- Describe the benefits of mindfulness
- Understand how mindfulness can be carried out for school children
- Be skillful to carry out a mindfulness session
- Describe the monitoring of changes in behavior due to mindfulness.

Session Outline (120 minutes)

Participants are all together for a discussion with the lecturer

i. Lecture (Power Point Presentation) (55 minutes)

ii. Practical session on mindfulness (60 minutes)

iii. Wrapping up the session. (5 minutes)

Material needed

- Power Point Presentation Slides
- Psychosocial Health Promotion of school children- A handbook for teachers
- Flip charts
- Marker Pens

- Mindful bell
- Toffees (twice as the number of participant)
- Transparent plastic bottle filled with water, gold dust and little amount of clear glue.
- Take home message sheet for each participant

At the beginning of the session all participants are seated together

I. Lecture (Power Point Presentation) (30 minutes)

The facilitator would deliver the lecture on mindfulness and mindful school to the participants. All participants would follow the lecture. Questions would be welcome at the end or during the session in-order to clear out participant doubts. The facilitator would encourage questions both during and after the presentation. The power point slides are attached herewith.

II. Practical session on mindfulness (60 minutes)

The facilitator would carry out a session on mindfulness within the class in-order to give the participants a first -hand experience on how to carry out a session on mindfulness. The facilitator should carry out one or more of the following mindfulness sessions.

i. Mindful Sitting
ii. Mindful Walking
iii. Mindful Listening
iv. Mindful Eating
v. Mindful Observing (observe the movements of gold dust withing the water bottle)

Following the practical session the 5 participants would be asked to share what they experienced and felt during the mindfulness session.

III. Wrap up the session (5 minutes)

Ask randomly selected participants on the key issues that were discussed during the session. Thereafter the participants could fill in their take home message form.

Chapter 17; Mindful School (Sati Pasala)A Newly Recognized Intervention to Improve Psychosocial Wellbeing

1. ……………………………………………………………………………………….........

   ……………………………………………………………………………………...…

2. ……………………………………………………………………………………..……

   ……………………………………………………………………………….…….…

3. …………………………………………………………………………..…………

   ………………………………………………………………………..……………

4. ……………………………………………………………………..………………

   ……………………………………………………………………………………………

5. ……………………………………………………………………………………………

   ……………………………………………………………………………………………

Sesssion

18

Training module based on the contents of Chapter 18; Multi Sectoral approach to improve psychosocial health of school children

This session aims to provide the participants with an overall understanding on the importance of the multi sectoral approach to improve the psychosocial health of school children.

Objectives

At the end of this session, participants should be able to

- Understand how mental health contributes to provide a child good health in totality
- Understand how mental health can be promoted in home, School, Living place – village/town, Government offices, Private institutions, Public transport services, School transport services, Sports ground/ stadium

Session Outline (70 minutes)

i. Introduction by the facilitator (5 minutes)

ii. Read pages 143-144 of the psychosocial guide (5 minutes)

iii. Group Discussions (55 minutes)

iv . Wrap Up Session (5 minutes)

Material needed

- Psychosocial Health Promotion of school children- A handbook for teachers
- White Board
- Marker Pens
- Bristol Board
- Take home message sheet for each participant

At the beginning of the session all participants are seated together.

I. Introduction by the facilitator (5 minutes)

The participant would all be seated together. The facilitator would introduce the session by saying the following

"Childhood is the foundation of a physically, mentally, socially, mindfully and ethically fulfilled society.

A Prominent feature of the present social system is that it is more concerned about physical measurements. Nowadays parents and adults are more concerned in fulfilling child's physical needs like goods and foods. This happens within family as well as in the society, leading to a system of appreciation based on physical earns.

They are not concerned about their child's as well as their own psychosocial health.

Though it is so, it is a well-known fact that feelings such as kindness, love, peace, companionship are necessary to maintain good relationships.

Therefore it is very important to ensure that our children have a healthy mental health atmosphere at all places. Through this session we will see how we could ensure this."

II. Read pages 143-144 of the psychosocial guide (5 minutes)

Thereafter the participants would be asked to read out aloud the contents of pages 143-144. The facilitator may choose the readers.

III. Group Discussions (55 minutes)

Thereafter the participants would be asked to count from 1-7. All those who counted 1 would be allocated the school, those who counted 2 would be allocated the living place, those who counted 3 would be allocated the government offices, those who counted 4 would be allocated private institutions those who counted 5 would be allocated public transport services, those who allocated 6 would be allocated school transport services and those who counted 7 would be allocated the sports ground.

Each group would discuss and plan how they could contribute to bring about good mental health among children in each of these places. They would document their strategies on a Bristol board.

They can refer the allocated part from the psychosocial guide.(from page144-151)

Following the group discussions the facilitator would get together two large white boards.

He will mark a space for each of the groups on the white board. On the center he will write in big block letters "THE MULTISECTORAL APPROACH TO IMPROVING THE PSYCHOSOCIAL HEALTH OF SCHOOL CHILDREN."

The facilitator would then ask all the groups to put up their Bristol boards on the white board in the allocated space.

After that one person selected by the group would present the strategies that the group came up with that should be in place to ensure the psychosocial health of school children.

IV Wrap Up Session (5 minutes)

Ask randomly selected participants on the key issues that were discussed during the session. Thereafter the participants could fill in their take home message form

Chapter 18; Multi Sectoral approach to improve psychosocial health of school children

1. ……………………………………………………………………………………………

   ……………………………………………………………………………………………

2. ……………………………………………………………………………………………

   ……………………………………………………………………………………………

3. ……………………………………………………………………………………………

   ……………………………………………………………………………………………

4. ……………………………………………………………………………………………

   ……………………………………………………………………………………………

5. ……………………………………………………………………………………………

   ……………………………………………………………………………………………

Taking a History of a Presenting Problem / Concern from an Adolescent

The HEADSSS assessment is a widely used psychosocial assessment which addresses health risk behaviours and resilience factors for young people.

The way in which you approach the young person may help to establish a good rapport for the rest of the consultation.

- Greet the adolescent first
- Ask the young person to introduce the rest of the family
- Begin by seeing the young person on their own and then bring in the family.

A major advantage of seeing young people on their own is that increases the chance that we can understand their perspective, concerns and agenda.

In some circumstances it may be appropriate to see the young person with their parents/carers first and then to see the young person alone (for example when the young person has had a seizure or collapse and third party witness account is required.)

Rarely, you might need to speak with the parents alone before speaking to the young person. In such cases ensure the teenager understands why this is necessary.

Assuring Confidentiality

It is essential to assure young people that the content of your conversation will remain confidential, and that you will not discuss things with their parents. “Anything we talk about today is confidential. That means I cannot tell others, including your parents, about it without your permission.Once you built up the rapport and found out that the young person is at risk of harm (eg. Physical/Sexual abuse or self harm)or if others would be at risk of serious harm, then you should explain the young person and get permission to discuss the matter to a very close and trust worthy adult of the young person.

Building rapport

This can be done by:

- Asking developmentally appropriate questions and avoid medical jargon
- Open ended questions for older adolescents
- Give alternatives for younger teenagers (eg which do you prefer maths or PE)
- Start with non-threatening topics (for example, if self harm is the presenting problem start with some questions about home or school)

- Listening to the teenager and giving them the chance to tell their story (resist the urge to jump in too soon to clarify)
- Take their concerns seriously – do not minimize their concerns
- Avoid lecturing teenagers – criticize the activity not the young person
- Minimize note-taking during the consultation
- Try not to make assumptions based on limited evidence (for example that a sexual partner is of the opposite sex)

The HEEADSSS Assessment

Home and relationships

- Who lives at home with you? Do you have your own room?
- Who do you get on with best/fight with most? Who do you turn to when your feeling down?

Education and employment

- Are you in school at the moment? Which year are you in?
- What do you like the best/least at school? How are you doing at school?
- What do you want to do when you finish?
- Do you have friends at school? How do you get along with others at school?
- Do you work? How much?

Eating

- Are you worried about your weight or body shape?
- Have you noticed any change in your weight recently?
- Have you been on a diet? Do you mind telling me, how?

Activities and hobbies

- How do you spend your spare time? What do you do to relax?
- What kind of physical activities do you do?
- At this stage - reassure about confidentiality

Drugs, alcohol and tobacco

- Does anyone smoke at home?
- Have you been offered cigarettes? How many do you smoke each day?

- Have you tried or been offered alcohol? How much/how often?
- Have you tried any other addictive substances? How much/how often?

Sex and relationships

- Do you have any intimate relationship with anyone at the moment? Are they a boy or girl?
- Young people are often starting to develop intimate relationships? How have you handled that part of your relationship?
- Have you ever had sex? What contraception do you use?
- Self harm, depression and self image
- How is life going in general?
- Are you worried about your weight?
- What do you do when you feel stressed? Do you ever feel sad and tearful?
- Have you ever felt so sad that life isn't worth living? Do you think about hurting or killing yourself?
- Have you ever tried to harm yourself?

Safety and abuse

Do you feel safe at school/at home? Is anyone harming you? Is anyone making you do things that you don't want to? Have you ever felt unsafe when you are online or using your phone?

Annex II

Psychosocial Health Promotion among School Children

What you can do as TRAINERS in Creating environments that promote and sustain positive psychosocial health for everyone

1. Build Healthy Public Policy

1. Encourage schools to have a written School Health Policy and display it
2. Always encourage and promote 13 year compulsory education
3. Improve the quality and coverage of School feeding programme
4. Allow and encourage students to participate in decision making; encourage teachers to do so
5. Empower teachers by providing alternatives disciplinary measures to corporal punishments to allow prospering school connectedness
6. Promote /encourage schools to have policies that contribute to the physical and emotional safety of all students
7. Encourage establishment of school Health club and school Health Advisory Committee
8. Encourage the schools to value all children- You – be the example
9. Provide models and mechanisms to regularly practice relaxation exercise in and out of class rooms
10. Help to establish counselling services within the school and closely monitor. Support the process when and wherever they need help; make yourself accessible when they need help!

2. Create Supportive Environments for Health

1. Actively support schools to convert their schools to a Health promoting school
2. Encourage teachers and staff to have a physical and psychosocially healthy classroom environment (child Friendly school)- provide them with necessary knowledge and skills
3. Encourage fostering an atmosphere of trust, tolerance, co-operation and empathy
4. Promote /encourage teachers and parents to showcase student's achievements / efforts and unity (not limiting to academic achievements)
5. Help the school to design physical spaces so that students can access facilities, and participate fully in planned learning activities
6. Encourage /support to provide a religious environment at school & at home

3. Strengthen Community Action for Health

1. Encourage teachers to interact with the parents regarding student learning issues
2. Collaborate with parents in improving psychosocial environment in school
3. Encourage /Support schools to establish and maintain a good relationship with the community
4. Offer opportunities to students /schools work with the community around the school in health related /non health activities

4. Develop Personal skills

1. Promote skills based education to develop personal skills
2. Promote Life skills programme for grade 6 and above & support to conduct such programmes
3. Support /strengthen SRH education for students and Comprehensive SRH for grade 12 and above
4. Have a Special focus on developing Coping skills and resilience among students
5. Help students for Self-identification of emotions
6. Always encourage promotion of physical activities at school and at home
7. Help schools to develop and conduct programmes on Mindfulness

5. Reorient Health Services

1. Always make an extra effort to do a quality and comprehensive school medical inspection which not only focus on physical health but also psychosocial health
2. Try to offer the best options in managing the identified health problems: ALWAYS think about the "Best interest of the Child"
3. Promote /engage research to increase evidence based interventions for school health
4. Establish and sustain Youth friendly health services- clinics; make continued efforts to advertise the services available – in the schools /community using multiple communication methodologies. These clinics are available in all Medical Officer of Health offices and some hospitals. For further details inquire, "www.yowunpiyasa.lk"
    - Publicize services available including 24 hour hot line services- e.g. Health Promotion Bureau, Sumithrayo, Mal Medura

Annex III

Activities for School Children

Session 1- Adolescent Brain Development

Part 1

Maturation of brain cells start from birth and continues until 25 years. It starts from back of the brain. Around 15 years, myelin is deposited over the amygdale, which perceives emotions.

Therefore, adolescents do take up risk behaviors emotionally, (e.g.: fast driving, violence, abuse, self-harm, suicide). They can't control their emotions due to immature frontal lobe, which is important in decision making. (Grganigy and Manning) Most of the time, adolescents take decisions without thinking about the consequences.

Brain takes nearly 25 years to mature

"Amygdale" the emotional center at the base of the brain, is fully mature by the age of 15.

Thus, a 15 year old youth experiences same emotions as his parents or rest of the adults around.

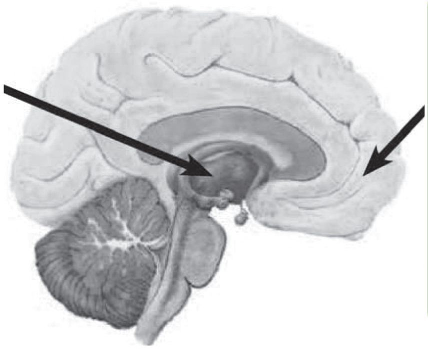

The **"emotional control center" based at the frontal cortex of the brain is still growing and myelinating.** When coupled with inexperience, it is no wonder why these youths have no grip on their emotions.

Part 2

Give the students an opportunity to involve in the following activities.

1. Planning and organizing various school programmes/ events

   Eg: Annual sports meet/ Science day/ Annual school trip
2. Making policies for the school

   Eg: Anti bullying policy, Health promoting school policy

Session 2- Life skills

Tell them they are going to learn about important skills that will be of importance in every sphere of life.

Activity 1

Write down the following professions in the board.

Cook, Driver, Teacher, Nurse, Dancer, Babysitter, Student, Traffic police, and Carpenter

Discuss the skills required for these professions with the students.

Guidelines for the facilitator:

You can add more jobs to clarify the concept of skills -like a computer operator, gardener etc.

- Literacy skill includes reading, writing, and arithmetic.
- Functional skill includes work, which helps in general functions and does not require any training.

  e.g. getting a letter registered.
- Livelihood skill includes work done in order to earn a living.
- Professional skill includes skills to be acquired by undergoing training in a specific field.

Activity 2

Divide the class into small groups.

Now ask them "Why are some people better at their work than others?" Let the participants brainstorm & note down their responses.

Expected Responses: they work harder, they have inborn talent, they learn, they are skilled, talented, God -gifted, face challenges well, etc

Guidelines for the facilitator:

Tell them that the people who are good at their task are called skilled or experts (give them some good examples of skilled people like football/cricket player, actor, surgeon, musician, dancers etc) To be skillful means to be capable at doing something well. Tell them that, just as the way these people succeed because they are skilled, all of us can succeed in life by consciously using life skills. We all can lead happier, healthier and successful lives by using the life skills. Tell them this introductory lesson to life skills will in turn form the basis for the future lessons.

Activity 3

Tell them that we have been able to differentiate betweenskills. But there is one more section of skills -very different from these- called "LIFE SKILLS". Tell them that our life depends on learning these Life Skills (LS) very well and applying them in our day to day challenges.

Ask the participants to give examples of a few other skills which are not basically meant to earn livelihood but still are important for a good life.

You may initiate by giving hints like: Don't we require to- Solve our problem? , Reconcile our differences? Resolve our conflicts? Be understood? etc. These require the use of Life skills.

Put up the definition of "Life Skills" on display using either chart or black board.Ask one or two students to read it twice.

Definition of Life skills:

Life skills are abilities for adaptive and positive behaviour that enable individuals to deal effectively with demands and challenges of everyday life.” (World Health Organization - WHO)

Activity 4

Put up the following figure for display using either chart or black board. Read out the names of the skills and explain the three broad categories of skills and the 10 core life skills. Explain the life skills. Tell the students that all of us use these skills in one way or the other but we need to learn to use them consciously and effectively

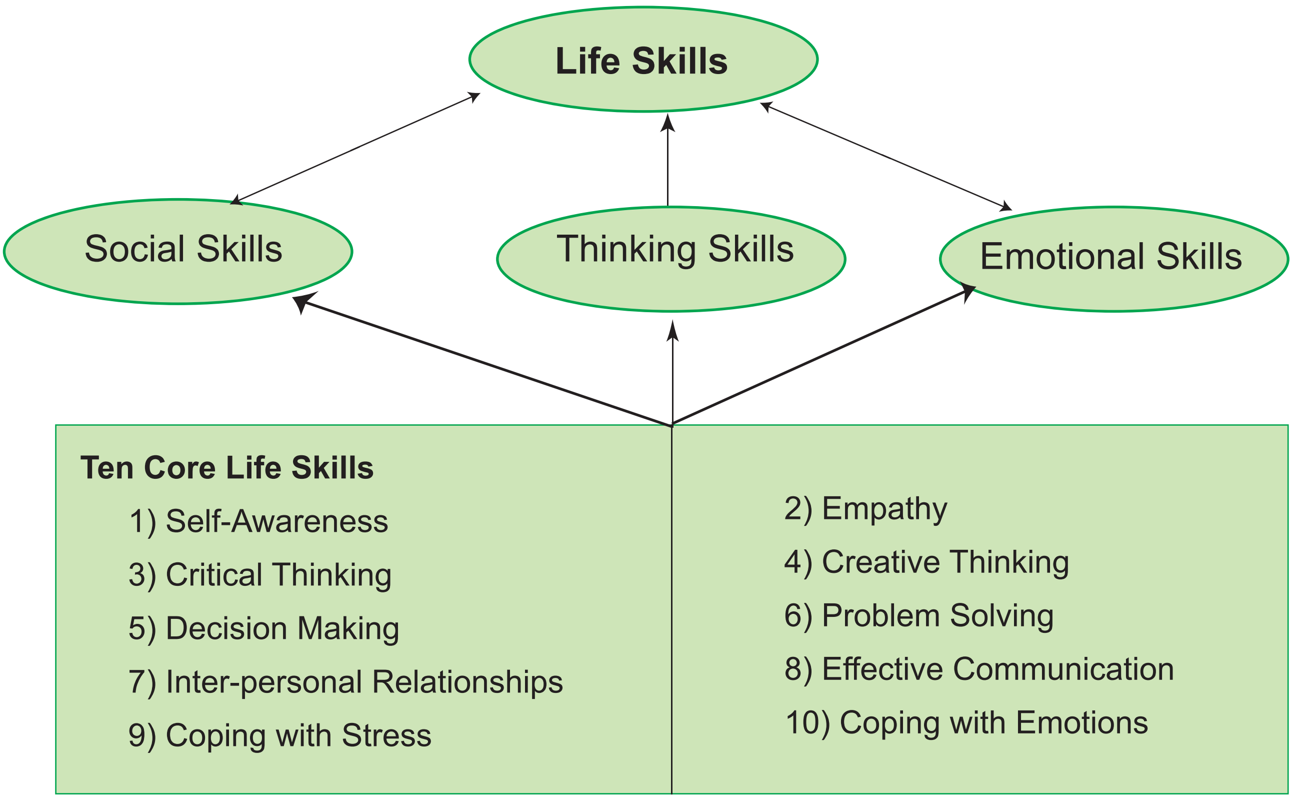

Ten Core Life Skills

1) Self-Awareness
2) Empathy
3) Critical Thinking
4) Creative Thinking
5) Decision Making
6) Problem Solving
7) Inter-personal Relationships
8) Effective Communication
9) Coping with Stress
10) Coping with Emotions

Thinking Skills	Creative Thinking
	Critical Thinking
	Decision Making
	Problem Solving
Social Skills	Self-Awareness
	Effective Communication
	Interpersonal Relationship
	Empathy
Emotional Skills	Coping Skills
	Coping with Stress

Guidelines for the facilitator:

It is best to explain about each skill by using simple language and by giving examples. For example – explain "Empathy" as "putting oneself in other person's shoes", Self-Awareness as "Knowing more about one's own self", Effective Communication as" capability of transmitting and receiving messages effectively" etc. Ask students to give some examples.

Activity 5

Ask students to list out "How learning about LS will help me!" in their notebooks.

Expected responses

Effective Acquisition of Life Skills Can Influence

The way we feel about ourselves, know our strengths and weaknesses

The way others perceive us

Our productivity

Self-confidence

Self esteem

Interpersonal relations and connectedness

Being more creative in problem solving

Correct decision-making

Ability to work in a team

Activity 6

Ask students to list out what LS would be required to choose an appropriate gift for their best friend on her/his birthday.

Expected responses: Creative Thinking, Critical Thinking, Decision-Making, Interpersonal Relationships, Empathy

Session 3 - Happy home environment

Activity 7 – Developing "web of concern" and "web of effort"

Ask students to identify eight areas of concern at home e.g.

A - Healthy and attractive environment

B - Opportunities gained to do event organizing

C - Time spent with friends

D - Time spent with parents

E - Time spent with siblings

F - Opportunities gained to talk to the teacher

G - Bullying free environment

H - Violence free environment

Ask the students to draw a circle with diameter of 20cm. Tell them to divide it in to eight parts as illustrated. The students should write their concerns against each line i.e. A to H (as above)

Each line is now divided into **10 parts** and numbered – 0 at the center and 10 at the circumference as illustrated.Ask the students to rate how important each concern is to them – from 1 to 10. Mark that point on the line and join all the points with a **green** pencil. E.g. Sehas might grade concern D with a level of 10, concern B with 8 and so on. This is the **'Web of Concern'.**

Next, ask students to **rate themselves** on a scale from 1-10 against each concern, indicating how much effort they are actually taking at home. Let students indicate these on the lines and join them upwith a **red** pencil. This is the **'Web of Effort'.**

Guidelines for the facilitator:

An example of the illustration has been given here.The students have already identifies their concerns and these may be the same or different from those illustrated. Tell the students that each student will be doing this activity alone. If desired a student need not even share her work with neighbours while doing it. Each student should rate each concern sincerely and also see how much she is doing to solve the issues.

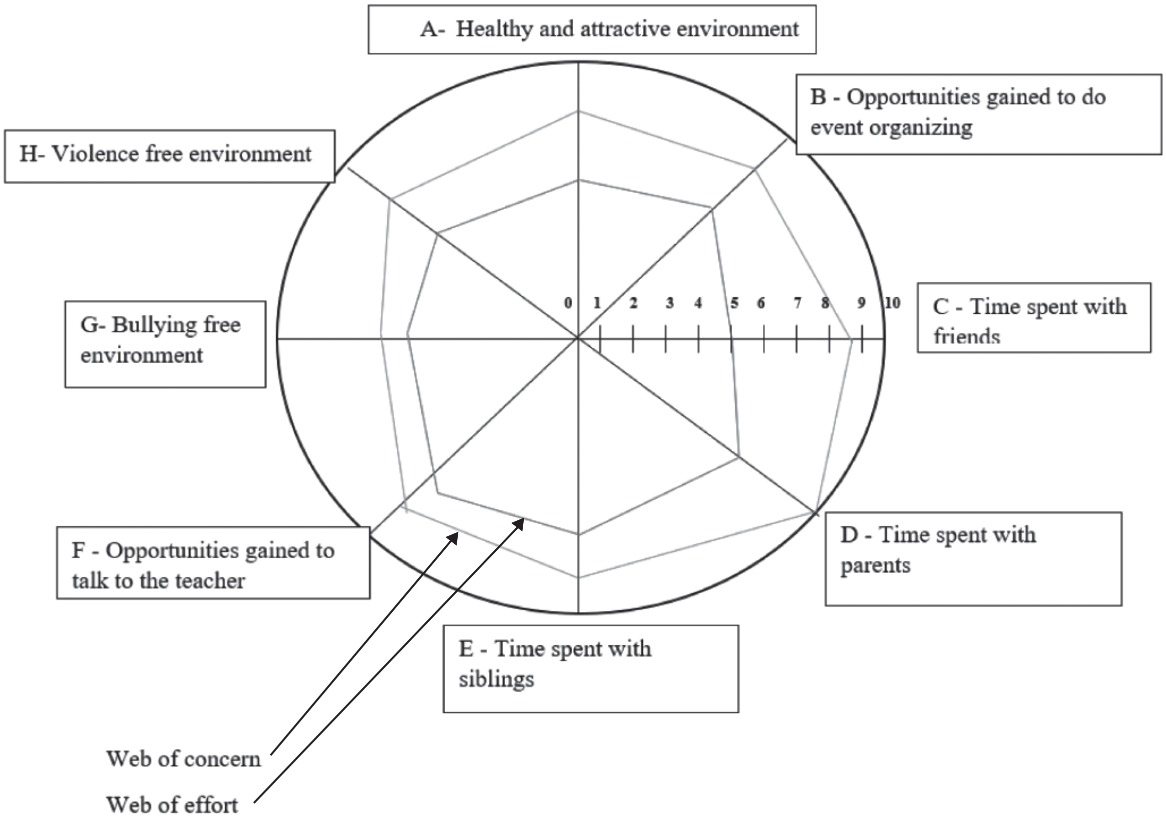

Now, ask students to compare their web with their neighbour's web.

Ask "Is there a gap between your web of concern and your web of effort? Why?"

Ask "How can we bridge this gap?"

Session 4 - Stress and Educational Problems in Adolescence

Activity 8

Write the word **"DESSERTS"** on the board, and ask a student to read it.

Now ask the class what this means?

Possible Responses:

sweets, tasty, yummy, ice cream, cake, pastry etc

Let them laugh and enjoy.

Lead them to say words like good, happy by asking "How does it make you feel?"

Now ask someone to read it in a reverse fashion – i.e. "STRESSED".

Ask "How does this make you feel?"

Possible Responses: Tense, angry, anxious, depressed

Now introduce the topic and ask the students, "What do you understand by stress?"

Note down their responses on the board.

Possible responses:

Tension

Tiredness

Anxiety

Irritation

Fatigue

Nervousness

Fear

Activity 9

Distribute four different colour cards to each student. Write Events on colour A,Physical on colour B, Emotional on colour C and Behavioural on colour D.

Ask them to think critically about

- the events that have caused them stress. Ask them to write three such events on the first colour A card (Stressors)
- What happens physically to their body when they are stressed? on colour B card(Physical)
- How do they feel mentally when they are stressed? On colour C card. (Emotional)
- What do they do when they are stressed? on colour D card(Behavioural)

Ask them to paste the cards on the newspapers on the wall in four raws – all cards of the same colour in each row.

Call on some volunteers one by one and ask them to read out ther responses.

Ask the class to have a look at all the cards and see the impact of stress on our bodies, feelings and behaviour.

Note the responses and ensure that the students are clearly able to categorize physical, emotional and behavioural effects of stress.

Possible responses

Stress-causing events (Colour A)

When my mother scolded me

When I have a test the next day

When my friend doesn't believe me

When I had to go alone to my friend's house.

Effect on the body (physical) (Colour B)

Headache, grinding teeth, shivering, trembling, fever, body ache, loss of appetite, breathlessness

Effect on feelings (emotional) (Colour C)

Depressed, angry, irritated, anxious

Effect on behaviour (behavioural) (Colour D)

Do not sleep, eat a lot, do not eat, cry, break things, shout at others.

Now ask them "What impact will such stress have on us and our work output?"

Possible responses:

Depression

Irritability

Extra tiredness

We will not have proper concentration

Poor performance in exams **(but mild stress can improve work performance)**

Panic attacks

People start taking drugs

Harm ourselves

We can start getting ill often

Since prolonged stress can impact on your health, it is important to develop positive coping mechanisms to manage the stress in your life.

Explain to the students that many events / factors in life can lead to stress. Tell them that the feeling of stress is partly due to **external** factors, but mostly due to **internal** factors which are **under our control**. In other words the events that happen may or may not be under our control but how we react to them is definitely under our control.

By utilizing **Life Skills** of **Critical Thinking, Self Awareness, Creative Thinking** (including humour), **Problem Solving, Interpersonal Relationship** etc we can overcome or manage stress. This is one very good example where a combination of Life Skills can assist another life skill.

Eg:

1. Critically analyze your work load and reduce unnecessary challenges.
2. Improve your relationships by doing team work.

Activity 10

Ask the students "Can there be anything positive about stress?"

Possible Responses:

- It can make us do better

Guidelines for the Facilitator:

Tell the students about Positive stress or **Eu- Stress** and negative stress or **Dys-stress.** Tell them that the situation remains the same. It is our response which decides our stress. If we respond in a positive fashion, we will excel, but if we respond in a negative fashion, we will get "stressed".

End the session by telling the students to convert their "Stressed" life into "Desserts" by converting negative behaviour into positive behaviour!

Is all stress bad?

No! To a certain extent stress is essential for our body to make it perform to the best of our ability.

This is called 'eu-stress'. Excess of stress leads to 'dys-stress'. At 'Optimum stress' we work at our best.

Stress is an integral part of our lives. If we are not under enough stress, then we may find that our performance suffers because we are bored and unmotivated. If we are under too much stress, then we will find that our results suffer as stress related problems interfere with our performance. It can lead to depression, suicide, substance abuse, panic attacks, etc.

It is important that we recognise that we are responsible for our own stress - very often it is a product of the way that we think. If we learn to monitor our stress levels, and adjust them up if we need to be more alert, or down if we are feeling too tense. By managing our stress effectively we can significantly improve the quality of our life.

It is important that adolescents are trained to cope with their stress. The first and most important step here is for adolescents to be able to recognize their stress, identify the situations that lead to stress and only then will they be able to manage it.

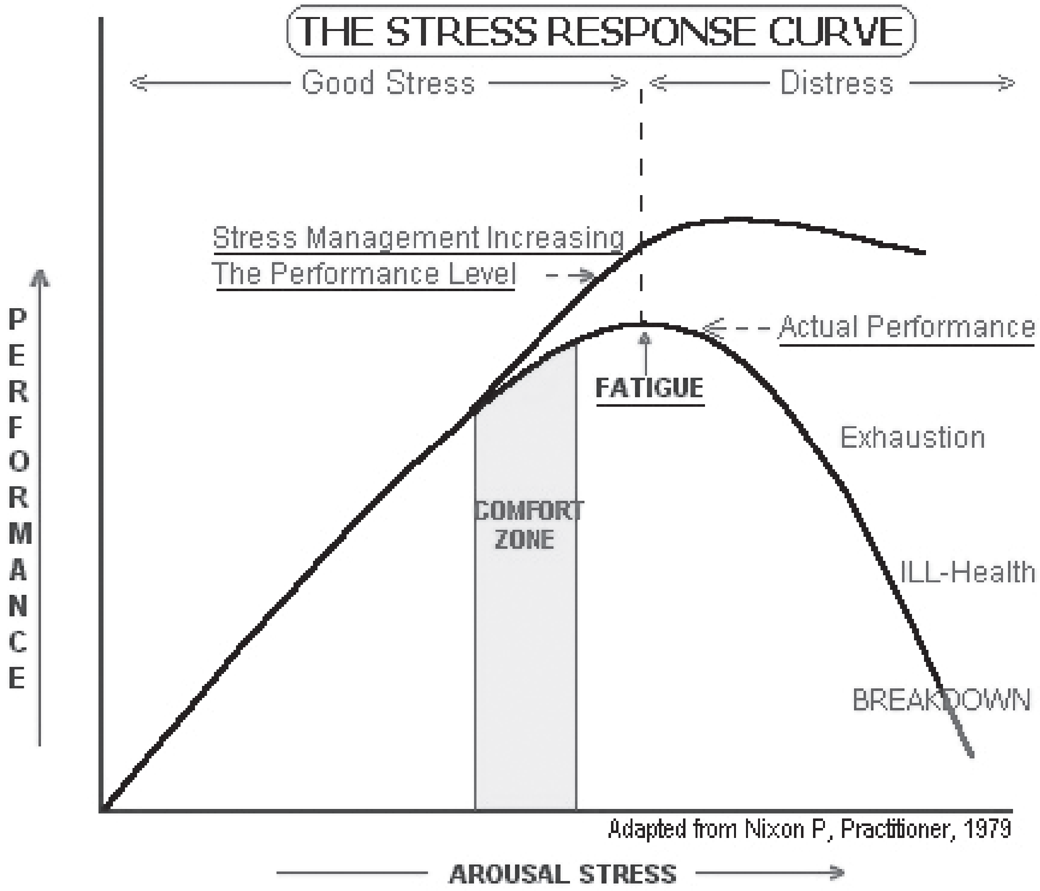

Awareness and acceptance have to precede the action and change in attitude.

Activity 11

Introduce ways of dealing with stress.

Eg: Relaxation techniques/ breathing exercises

Mindfulness practices (Please refer chapter 17 of Psycho Social Health Promotion of School Children A handbook for teachers)

Talking to a counselor / doctor trained in mental health

Session 5- Violence and bullying in schools

Activity 12: Agree – Disagree

1. Explain that you will read a statement about violence in schools. Each person should decide what they think about the statement. Do they agree, disagree, or aren't they sure? They should stand near the sign that shows what they think.
2. Read the first statement (use one from the box, or make up ones that will work with this group).Give them time to decide where to stand.

3. Ask a few people in each group to explain their opinion. Anyone else who hears something that makes them change their mind can move to a new place
4. Read the second statement: repeat step 3
5. Read the third statement: repeat 3
6. Ask the group:
   - How did you react to this activity?
   - What did you learn from it?
   - If you changed your mind- what made that happen?

Example statements

Physical punishment (eg: hitting/slapping) can be used if it helps children learn.

Verbal punishments (eg: name calling, humiliation) don't hurt children as much as physical punishments.

Children who are bullied or teased need to learn to defend themselves.

Activity 13 – Mapping

1. Divide the students into groups of four. Ask each group to draw a map of their school, or other places they go to learn or take classes, They may want to draw the school yard and streets near the school , too.
2. Have them mark on their maps places where violence takes place, and make a brief note to say what type of violence it is.
3. Hang the maps on a wall so the group can walk around and look at them
4. Discuss: How were the maps similar? How were they different?
5. On a large sheet of paper, write summary: What are the main types of violence? Where do they occur? How can they be prevented?

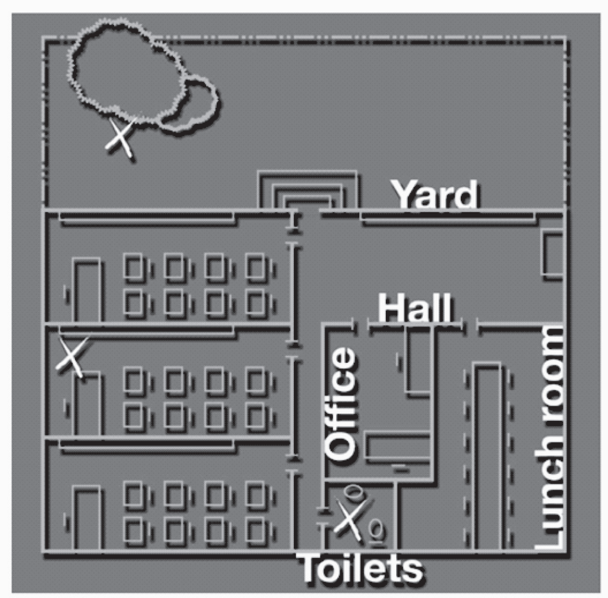

Activity 14

CREATING A WHOLE SCHOOL APPROACH TO ANTI-BULLYING

School communities are made up of a wide variety of people which include: students, teachers, supportive staff, catering staff, and parents. Adopting a whole school approach is important as it can affect any member of this community.

What is a whole school approach?

A whole-school approach is where every person who is part of the school, irrespective of their position, is not only aware of the school's approach to tackling bullying, but participates in its delivery. All attempts to adopt a whole school approach should require participation and involvement from every member of the school community; everybody has a role to play.

Appoint a team of anti bullying ambassadors (ABA) for the school

Anti Bullying Ambassador Team can use the planning tool (Students mapping tool) given below. This tool maps out 5 key areas that should get the ABA team thinking in an inclusive and equally ambitious manner to make this approach a success.

The whole school approach- Students mapping tool

Using the boxes provided, make your notes as an ABA Team on how you will address all 5 points of achieving the Whole School Approach

1. Having a vision for global learning:

What are you trying to ensure everyone knows in your school?

E.g. everyone know about online safety,promoting equality, how to report bullying incidents in the school, etc

2. Use reflections to plan further work by the ABA's:

what have you already done?
What do you want to do next and change for the better?

E.G. Rewrite the school's Anti-Bullying Policy to make it more child-centre, Conduct assemblies on the impact of bullying, mental health and other related topics, Develop staff training to be delivered by AntiBullying Ambassadors.

3. Connecting the work across different spaces within the school: e.g 1 school has grade 1 to 13, they have same policy and ABA practice across the whole school.

How can you do this in your school? Include hallways, classroom and staff room!

E.G. Anti-Bullying Team ensures that every person in the school has a copy of and is aware of the Anti-Bullying Policy. Noticeboards with the same content are in almost every school corridor.

4. Involving people across the whole community:

How do you go beyond the school premises so the community knows about your approach?

Eg:projects that can include parents local community groups, the nearby company's for fundraising events, parent workshops, dramas etc!

5. Embedding Anti-Bullying into the school culture:

How do you get all the school to be positive, accepting etc.

E.g. every class has to take part in one positive activity on designated days, Quizes on what bullying is, Promotion of the reporting procedure in whole school assemblies, Creating your own Upstander mission statement for all to sign up to

Session 6 - Effects of child abuse on childhood and adolescence

Sexual harassment can be requests for sexual favors or **unwelcome** sexual behavior that is **bad enough** or **happens often enough** to make you feel uncomfortable, scared or confused and that interferes with your schoolwork or your ability to participate in extracurricular activities or attend classes.

Activity 15

Ask the students, **"What are the different kinds of sexual harassment?" "Who can be the possible harassers?"**

Ask them to brainstorm on various types of sexual harassment. Note down their responses on the board.

Guidelines for the Facilitator:

Tell the class sexual harassment is defined as unwanted and unwelcome physical contact, verbal demands and put downs based on sex, gender or sexuality of the person being harassed.

Sexual harassment can be

verbal (comments about your body, spreading sexual rumors, sexual remarks or accusations, dirty jokes or stories),

physical (grabbing, rubbing, touching, pinching in a sexual way, sexual assault)

visual (display of naked pictures or sex-related objects, obscene gestures).

Sexual harassment can happen to girls and boys.

Sexual harassers can be family members, relatives, fellow students, teachers, principals, janitors, coaches, and other known people.

Activity 16

Divide the class into 3 groups. Give one copy of the quiz to eachgroup. Ask the students to examine each scenario in the quiz and decide which category they fall in:

a. Sexual harassment:

b. Unacceptable behaviour but not sexual harassment:

c. Acceptable behaviour

d. Unsure

Scenarios

1. A young man continually forces his girl friend to come to a hotel room with him and she always says 'no'. (a)
2. A young man shows a pornographic magazine to his male friends in front of a young woman. The man teases her about her body. (a)
3. The teacher gets angry and slaps a boy in her class. (b)

4. A young man threatens his girlfriend that if she doesn't have sex with him, he will tell his friends a nasty rumour about her. (a)
5. A male coach teaches tennis to a girl and holds her hand to teach her how to hold the racket. (c)
6. A group of young men and women often tease a woman they know has been raped. (a)
7. A young boy asks his friend to try marijuana. (b)
8. A group of young men often tease a young woman about the size of her breasts. (a)
9. A young girl asks a boy for his notebook. (c)
10. The "uncle" of a 16 year old girl kisses her on the lips forcefully. (a)

Ask the groups to answer the questions put to them at random. Correct their impressions where everrequired. Allow some discussion to take place.

Session 7 - Impacts of Information Technology and Media on Adolescents Safe use of media

Activity 17

Divide the class into 3-4 groups. Ask them to sit around the table.

Invite each group to nominate a leader.

The leaders then write the **discussion points** given below on small pieces of paper (prepared earlier).

Ask them to put them in a box placed in the centre of their group.

Discussion points: (Agree/Disagree)

1. TV has an adverse effect on studies.
2. TV should be watched only at a specified time.
3. It is essential to watch TV everyday.
4. TV affects our physical and mental health
5. TV should only be used as a means of information and knowledge.
6. TV promotes violence and areas like graphic sexuality.
7. TV has spoiled youngsters, especially students.
8. TV should be banned

Ask one student from every group to pick a topic from the box and decide whether he/she agrees or disagrees with the statement on hand.

Let him/her read it aloud to his/her own group and provide three reasons for agreeing or disagreeing.Others in the group make a note of their responses to the speaker's viewpoint which they will discuss at the end.

The next student from the group follows in a similar fashion.

The leader makes a note of the points that emerge. He/she ensures that the speech making is done without interruptions.Allow a brief discussion lasting five minutes or so.

Activity 18 - "Just for 30 seconds!"

After the debate within the group is complete, request the students at random to sum up the debate and highlight the key aspects of their discussion for the whole class.

State that the student from the group must speak non- stop for 30 seconds on the allocated point ("Just for 30 seconds!")

Risky online relationships

Give some examples of positive interactions between strangers online.

Sample responses:

- Selling your own products or possessions online
- Responding to job opportunities online
- Leaving comments on other people's blogs, even if you don't know them personally
- Playing games or interacting in virtual worlds with people you don't know offline

Give some examples of uncomfortable interactions between strangers online.

Sample responses:

- Dealing with awkward friend requests from people you don't know well
- Receiving mean or creepy comments from strangers
- Getting spam or junk mail
- Seeing instant messages from unknown screen names

Activity 19

EXPLAIN to students that they are going to read and analyze a story about a teen's risky online relationship.This story will challenge them to think beyond online predator stereotypes. They should pay attention to how the relationship develops, and be on the lookout for anything that seems like manipulation.

DIVIDE students into pairs or groups of three.

DISTRIBUTE the Maya's Situation Student Handout, one for each student. Give students 10 minutes to read the story and answer the questions on the handout in their groups.

INVITE groups to take turns sharing their answers to the questions on the handout.

Have all groups share their answers to the final question, which is about what advice they would give to Maya if they were her friend.

Maya's story

Maya is in grade 9 and during the school holidays she followed a computer course. She was an active student in this class and loved every minute of it. The course finished after one month. But now Maya misses the course and feels like she can't relate to her school friends anymore.

Every evening, Maya sends messages to Kishan, her computer instructor. He is in the university and lives a few hours away from her. Kishan and Maya didn't know each other that well, but they exchanged their Viber numbers at the end of the course. Maya talks to Kishan about how much she misses the course and why she's frustrated with her friends. Kishan is funny, flirty, and great at giving advice. "Those girls sound so immature," he tells her. "You might as well be in university. Seriously. You act way cooler than any 14-year-old I know."A few weeks after school starts, Kishan starts flirting with Maya more obviously:

Kishan Smart did you know i thought about you all the time during the computer course?

Maya Queen: why didn't you say anything!?

Kishan Smart lol u were my student

Kishan Smart… and i want to make sure u will still be a student in the next course….so we can't tell anyone about this…

Maya Queen :) ummm i better be your own student in the next course next holiday! jk. (but seriously.)

Kishan Smart parents freak out about this kind of stuff

Kishan Smart u don't want me to lose my job, right?

Maya Queen :) of course not!

Kishan Smart that's my girl. so glad that we can trust each other.

Kishan then asks Maya to send him some pictures of her in her swimming suit. Maya is flattered, but it makes her feel a little uncomfortable. She decides to send the pictures anyway. Kishan always talks about how mature she seems, and she doesn't want to make him think otherwise.

One day after badminton practice, Maya's best friend, Achini, asks to borrow her phone. Without trying to snoop, Achini sees a few sexts (sexually explicit text messages) between Maya and Kishan. Achini doesn't know much about Kishan, which is strange because she and Maya usually tell each other about their crushes. Achini decides to ask Maya about her relationship with Kishan.

Directions

Based on the story you just read, answer the following questions as if you were Maya. Write your responses in the spaces provided.

1. How did you meet Kishan, and how often do you talk to him?
2. Is Kishan older than you? By how much?
3. Has Kishan ever asked you to keep your relationship secret? In what way?
4. Have you ever felt uncomfortable with anything that Kishan has asked you to do?

Now answer the following questions as if you were Achini, not Maya.

1. Does it seem like Kishan is manipulating Maya? Why or why not?
2. Why might this be a sensitive issue for Maya?
3. What advice would you give Maya about her relationship with Kishan?

Guidelines for the Facilitator

Make sure the children answer the above questions based on Maya's story

The guide for the answers for Achini' s questions are as follows:

1. **Does it seem like Kishan is manipulating Maya? Why or why not?**

   Students may have mixed responses, but they should ultimately recognize that Kishan is being manipulative. He says things like "you don't want me to lose my job, do you?" and tells Maya that she's more mature than most girls her age. Maya might think that Kishan is just flirting with her. But it seems like Kishan has more influence in this relationship than Maya does, and he's guiding it to be both sexual and secret. Kishan also asked Maya to send him sexy pictures, which is risky. We don't know what will happen to those photos, and even if nothing does, Maya was uncomfortable with the request.

2. **Why might this be a sensitive issue for Maya?**

   Guide students to recognize that Maya misses the computer course a lot and doesn't feel like she can relate to her friends at school anymore. She likes talking to Kishan about computer course and she vents to him about her friend issues. Kishan flirts with Maya, too. She's flattered that someone older thinks she's mature and attractive.

3. **What advice would you give Maya about her relationship with Kishan?**

   Maya needs to know that her online relationship with Kishan is risky, and if she continues it, she could be in danger. Maya misses the computer course and is having a tough time with friends at school, and it's important to be sensitive to that. But to stay safe, Maya needs to realize that Kishan appears to be manipulating her, and she should cut off their relationship before it goes any further. Friends who are concerned about Maya's situation should also remember that they can, and should, seek help from a trusted adult if they think Maya is taking dangerous risks.

What Should Teens Know if Online Strangers Contact Them?

Teens should also reflect on these questions if they communicate with someone they meet online:

- Has this person asked to keep anything about our relationship a secret?
- Has this person hinted at or asked about anything sexual?
- Have I felt pressured or manipulated by this person?
- Do I feel true to myself – sticking to my values – when I communicate with this person?

If teens feel uncomfortable during a conversation with an online stranger, they should:

- Change it up. If something feels like it might be getting risky, it probably is. But if teens are not sure, they should try changing the subject, making a joke, or saying they want to talk about something else. If they still feel pressured or uncomfortable, they need to take further action.
- Log off or quit. Teens need to remember that at any time they can just stop typing and log off if a conversation gets uncomfortable online. They can also take action to block or report another user, or create a new account – whether for email, instant message or virtual world – to avoid contact with that person again.
- Know that it's okay to feel embarrassed or confused. It's not always easy to make sense of situations that make teens uncomfortable online. Nor is it easy for them to ask for help if they feel embarrassed about what they've experienced. They should know these feelings are normal.
- Talk to a friend or trusted adult. Teens should know that it's okay to reach out. Even if they feel they can handle a tricky situation alone, it's always a good idea for teens to turn to friends, parents, teachers, coaches,and counselors for support.

Session 8 - Conflict resolution and negotiation for better health

Activity 20

Greet the Students and introduce the topic.

Ask the class: **“What is Conflict?”**

Possible responses:

Two people/parties fighting over something

Tension, fight, anger, feeling of opposition, etc.

Conflict is defined as “the competitive or opposing action of incompatibles”.

Ask the class “How can conflicts harm our health and social standing?”

Possible responses:

Our peace of mind and happiness can be lost

We can get injured in fights.

We can do things in anger that can harm our social standing

There are three levels of Conflict:

Level 1: Can’t we just get along?

Level 2: I intend to win

Level 3: I will hurt you

State that conflict – either acute or chronic - can harm our health in many ways. It can snatch our happiness, joy and peace of mind. It can lead to violence against others and /or self. Chronic conflict situations can lead to hypertension, affect our heart and lead to depression. Some reports have also linked chronic stress to occurrence of cancers. In short, unresolved conflicts adversely affect our health– physical, mental, social and spiritual. They can also reduce or adversely impact our social standing and reputation.

Activity 21

State that it is important to learn how to reduce or resolve conflicts. Display the poster “**Possible Ways of Resolving Conflict”** prepared by you, or write the following on the black board.

Possible Ways of Resolving Conflict

Avoid conflict - Simply withdraw from any conflict.

Smooth it over - Pretend there’s no conflict and everything is **OK.**

Win at all Costs - Get what you want; the other person loses.

} Will lead to mor problems

Compromise - Give up something you want to get something else that you also want.<br><br>Win/win negotiation - Use creative problem solving to give both people what they want or need.	Successful methods

Ask:

Which methods usually cause problems?

Which options are better strategies? Why?

Give an example of a common situation in which two parties reach a compromise.

> **Guideline for the Facilitator:**
>
> Tell the Students: The first three situations given above usually lead to problems and the latter two are more successful. We will be doing activities to understand how to make/improvise a win/win negotiation.

Activity 22 - Role plays

Divide the class into 4 small groups and ask each group to think of a way of resolving the conflict in the given scenarios using a win/win option. Ask volunteers to develop a short role play which shows how to work out a win-win solution to this problem.

Scenario 1: Wasana wants to go to a movie with her friends on Saturday night. But she has been doing badly in school and her parents refuse to let her go out with her friends until she improves her grades.

Scenario 2: Pasan lives in a village and goes to school. He wants to go to the fair with his friends on the coming Sunday. But his parents tell him that they are expecting some relatives to come over on Sunday and they want him to help with work at home.

Activity 23

Display on Poster/Blackboard “Four Steps of Successful Win/Win Negotiation”: Ask avolunteer to read out the points.

Four steps of successful win-win negotiation

1. State your position. Use “I” statements, say what you want or need.
2. Listen to the other person’s position. Find out what the other person needs or wants. Restate the other person’s position to be sure that you understand.
3. Brainstorm WIN/ WIN solutions. Take into account both peoples’ needs and wants. Be creative.
4. Agree on a solution. Try it out. If it does not work, start the process over again.

Session 9 - Substance abuse

Implement the drugs prevention school programme as per the guidelines given by the Ministry of Education.

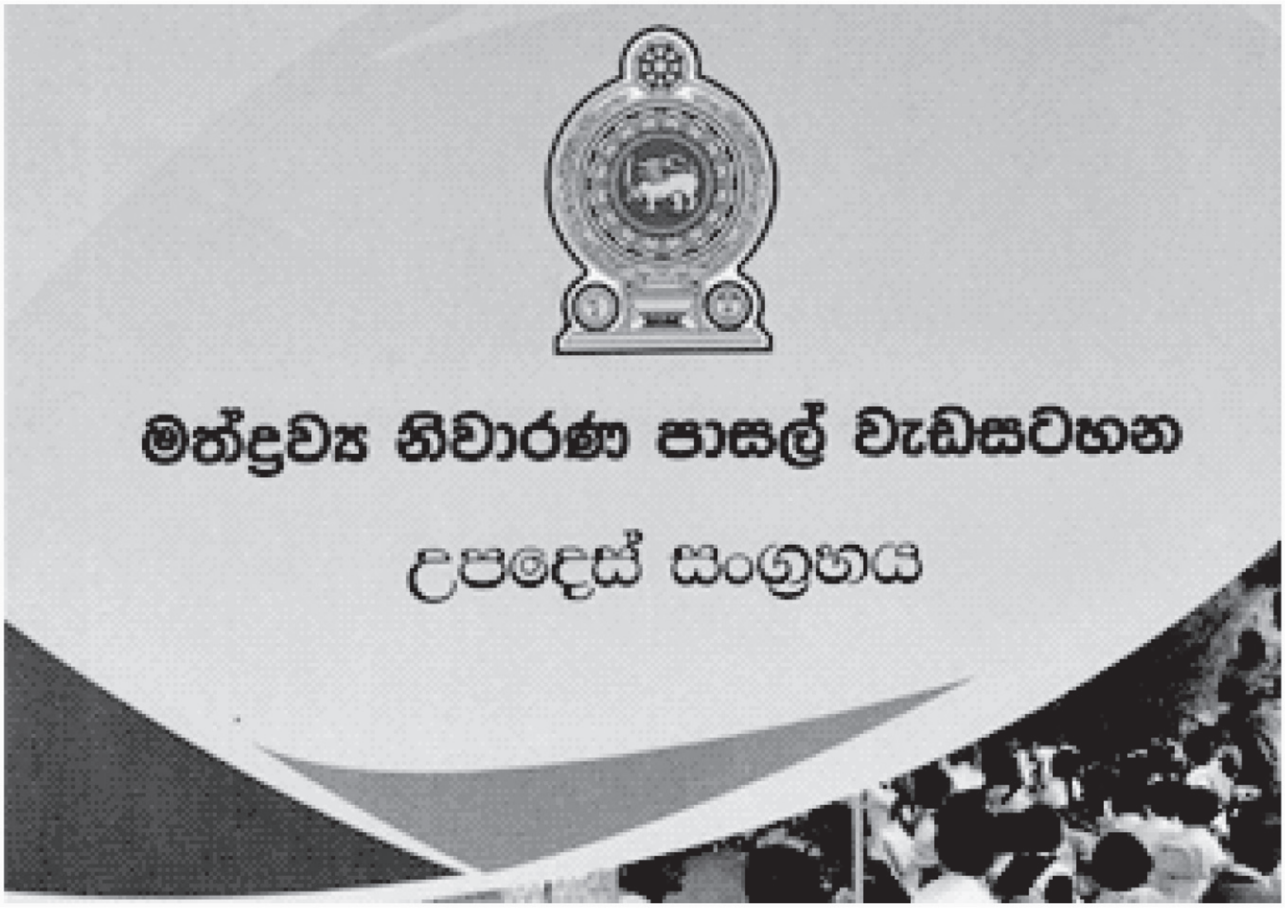

Supplement: Supplementary file 1 — Additional file 1. [file 12889_2023_17023_MOESM1_ESM.pdf]
